# USP35 promotes cell proliferation and chemotherapeutic resistance through stabilizing FUCA1 in colorectal cancer

**DOI:** 10.1038/s41389-023-00458-2

**Published:** 2023-03-03

**Authors:** Yi Xiao, Xiaoyu Jiang, Ke Yin, Tianshu Miao, Hanlin Lu, Wenqing Wang, Lijuan Ma, Yinghui Zhao, Chunyan Liu, Yun Qiao, Pengju Zhang

**Affiliations:** 1 Key Laboratory Experimental Teratology of the Ministry of Education, Department of Biochemistry and Molecular Biology, School of Basic Medical Sciences, Cheeloo College of Medicine, Shandong University, 250012 Jinan, Shandong China; 2 Eppley Institute for Research in Cancer and Allied Diseases, Fred & Pamela Buffett Cancer Center, University of Nebraska Medical Center, Omaha, NE 68198 USA; 3 Department of Pathology, Shandong Provincial Hospital, Shandong University, 250021 Jinan, Shandong China; 4 Department of Cardiology, Qilu Hospital of Shandong University, 250012 Jinan, Shandong China; 5 Department of Clinical Laboratory, The Second Hospital of Shandong University, No. 247 Beiyuan Street, 250033 Jinan, Shandong China; 6 Department of Integrated Traditional Chinese and Western Medicine, Medical College of Qingdao University, 266071 Qingdao, Shandong China; 7 Department of Traditional Chinese Medicine, Qilu Hospital of Shandong University, 250012 Jinan, Shandong China

**Keywords:** Colorectal cancer, Cancer therapy

## Abstract

Ubiquitin-specific-processing proteases 35 (USP35) is an under-characterized deubiquitinase and its role in colorectal cancer (CRC) remains unclear. Here, we focus on delineating the impact of USP35 on CRC cell proliferation and chemo-resistance, as well as the possible regulatory mechanism. By examining the genomic database and clinical samples, we found that USP35 was overexpressed in CRC. Further functional studies showed that enhanced USP35 expression promoted CRC cell proliferation and resistance to oxaliplatin (OXA) and 5-fluorouracil (5-FU), whereas USP35 depletion impeded cell proliferation and sensitized cells to OXA and 5-FU treatments. Then, to explore the possible mechanism underlying USP35-triggered cellular responses, we performed co-immunoprecipitation (co-IP) followed by mass spectrometry (MS) analysis and identified α-L-fucosidase 1 (FUCA1) as a direct deubiquitiation target of USP35. Importantly, we demonstrated that FUCA1 was an essential mediator for USP35-induced cell proliferation and chemo-resistance in vitro and in vivo. Finally, we observed that nucleotide excision repair (NER) components (e.g., XPC, XPA, ERCC1) were up-regulated by USP35-FUCA1 axis, indicating a potential mechanism for USP35-FUCA1-mediated platinum resistance in CRC. Together, our results for the first time explored the role and important mechanism of USP35 in CRC cell proliferation and chemotherapeutic response, providing a rationale for USP35-FUCA1-targeted therapy in CRC.

## Introduction

Colorectal cancer (CRC) is a prevalent and lethal malignancy worldwide [1]. It is the third most commonly diagnosed cancer and the third leading cause of cancer-related mortality in men and women in the United States [2]. Although CRC is a detectable and curable disease if diagnosed at an early stage, nearly two-third of the patients is diagnosed at advanced stages with a stark decrease in 5-year survival rate [1–3]. For the local CRC, endoscopic and surgical removals are the mainstay treatments, whereas for the regional and metastatic cases, surgery as well as systemic treatment, including adjuvant chemotherapy, targeted therapy, immunotherapy, radiotherapy, etc. are required [3]. Of note, identifying critical cancer targets for targeted therapy has been of tremendous interest in the biomedical field over the past two decades, aiming to increase the specificity in cancer treatment and overcome drug resistance of the traditional regimens [4]. In the case of CRC, new options of targeted therapy have been unceasingly revealed, bringing great therapeutic avenue for the cancer patients [5]. Therefore, unraveling novel targets in CRC will offer new opportunities in improving treatments for the CRC patients.

Ubiquitination is a common post-translational modification that by affecting protein stability, interaction, localization, and activity, regulates fundamental biological process, including cell division, fate specification, migration, etc. [6, 7]. Aberrant protein ubiquitination due to altered expression or activity of ubiquitin-activating enzyme (E1), ubiquitin-conjugating enzyme (E2), ubiquitin ligase (E3), deubiquitinase (DUB), and proteosome, contributes to cancer development [6, 8]. Drugs targeting the ubiquitin–proteasome system (UPS) components have achieved substantial progress in cancer treatment, with several proteasome inhibitors approved by the Food and Drug Administration (FDA) and many other inhibitors actively tested in pre-clinical studies [8, 9]. For example, the catalytic core of the 26S proteasome complex, S20 proteasome, is targeted to disrupt cancer proteostasis, with three renowned drugs approved by FDA (e.g., bortezomib, carfilzomib, and ixazomib), especially in treating multiple myeloma [10]. The immunomodulatory drugs (IMiDs; e.g., thalidomide, lenalidomide, and pomalidomide) targeting E3 ubiquitin ligase complex component cereblon (CRBN) are another group of drugs approved by FDA, that by binding with CRBN, switch the substrate specificity, and ultimately lead to the degradation of critical pro-survival proteins in multiple myeloma [11]. Moreover, some USP-targeting drugs, such as MLN4924 (NEDD8 inhibitor), TAK-243 (UAE inhibitor), KSQ-4279 (USP1 inhibitor), etc., are or will be examined in the clinical trials, and many other inhibitors are proved to have great therapeutic potential in pre-clinical studies [8]. DUBs function to catalyze the removal of ubiquitin (Ub) moieties from targeted proteins, and accumulating evidence has suggested that DUBs are attractive therapeutic targets owing to the well categorized catalytic domains [12–17]. Up to now, there are roughly 100 identified human DUBs, and the characteristics of many DUBs are still unknown [13]. Here, we aim to understand the role of one of the under-characterized DUBs, ubiquitin-specific-processing proteases 35 (USP35), in this study.

USP35 is a member of the cysteine proteases C19 family [18]. Recent works have suggested that USP35 is a promising cancer target due to its role in tumor growth and chemo-resistance by regulating the stability of important players in tumor development and cell death [19–23]. For example, USP35 promotes tumorigenesis in estrogen receptor (ER)-positive breast cancer by stabilizing and activating estrogen receptor α (Erα) to increases Erα transcriptional activity [19]. USP35 resists cell apoptosis by stabilizing anti-apoptotic factor BIRC3 and relieves endoplasmic reticulum stress (ERS) by deubiquitinating RRBP1 [20, 22]. USP35 also reduces ferroptosis by enhancing ferroportin-mediated iron export [21]. Moreover, USP35 suppresses the induction of type I interferons by interfering with STING-TBK1-IRF3 pathway [23]. Despite the potential role of USP35 in tumorigenesis and drug resistance, there is still a knowledge gap concerning the role of USP35 in CRC. Hence, we focus on investigating how USP35 affects carcinogenesis and drug resistance in CRC.

Altered glycosylation is a common feature in cancer and of great clinical significance in cancer screening, diagnosis, targeted therapy, and prognosis [24]. In our journey to find the possible mechanism, through which USP35 exerts its role in CRC, we identified α-L-fucosidase 1 (FUCA1) as a potential target. FUCA1 is an enzyme that hydrolyzes terminal fucose residues from glycolipids or glycoproteins [25, 26], and its function in cancers remains obscure and controversial. While a few studies suggest that FUCA1 restrains tumor growth, attenuates cell motility, triggers cell death, and sensitizes cancer cells to chemotherapy [27–29], two other studies lead to quite opposite conclusions [30, 31]. Therefore, we performed a series of functional studies under the regulation of USP35-FUCA1 axis, aiming to better understand the role of FUCA1 in cancers.

In the present study, we first demonstrated that USP35 promoted CRC cell proliferation and resistance to the drugs (oxaliplatin and 5-fluorouracil) routinely used in the CRC clinic. Then, we performed co-immunoprecipitation (co-IP) followed by mass spectrometry (MS) analysis, and identified FUCA1 as a potential target of USP35. We further confirmed that FUCA1 was deubiquitinated and stabilized by USP35. Additionally, we showed that the impact of USP35 on CRC cell growth and chemo-resistance was mediated by FUCA1 in vitro and in vivo. Finally, we suggested that USP35-FUCA1 axis up-regulated nucleotide excision repair (NER) components (e.g., XPC, XPA, ERCC1), which could be a potential mechanism for platinum resistance in CRC. Overall, this study indicates that USP35 contributes to tumorigenesis and confers chemo-resistance in CRC by deubiquitinating FUCA1, unraveling a novel molecular target for CRC treatment.

## Materials and methods

### Cell culture and transfection

HEK293T and the CRC cell lines HCT116 (wild-type p53), LoVo (wild-type p53), DLD-1 (S241F-mutated p53), and HT29 (R273H-mutated p53) cells (Table S1) were purchased from the Cell Bank of the Chinese Academy of Science (Shanghai, China) and cultured following the instructions. Cells were resuscitated every 3 months and tested negative for mycoplasma contamination.

Polyethylenimine (PEI, Polysciences, Inc.) was used for transfection. psPAX2 and pMD2.G (GeneChem Co.) were used for lentiviral packaging. RFect (Changzhou Bio-generating) was used as transfection reagent. The transfection was conducted following the manufacturer’s instructions.

### Expression constructs and RNA interference

Human wild-type (WT) USP35 vectors pCMV-3X-HA-USP35/HA-USP35 and pCMV-3X-Myc-USP35/Myc-USP35, and the catalytically inactive USP35 mutant pCMV-3X-HA-USP35 C450A/USP35 C450A were previously constructed by our laboratory [22, 32]. Human FUCA1 vector pcDNA3.1-Flag-FUCA1 was purchased from Fenghui Biotechnology Co. USP35 cDNA was cloned into the pLVX-IRES-Puro vector (Addgene) for lentivirus production. The specific shRNAs was cloned into the pLKO.1-TRC vector (GenePharma) and the targeting sequences are: shUSP35-1: 5’-GCTGAGTTGGGCTCTTCTAGA-3’; shUSP35-2: 5’-GCGTCTGACTTCAGACATTG-3’; shFUCA1-1: 5’-CGCAGAGTTTGCTTGGACTAT-3’; shFUCA1-2: 5’-GCAACTATCTTCTGAACATTG-3’; shFUCA1-3: 5’-GGAAATGGCTGAGCATCAATG-3’; shFUCA1-4: 5’-GGTCCACAGATCCAGATAATT-3’. The non-effective scrambled shRNA targeting sequence is 5’-GTTCTCCGAACGTGTCACGT-3’. The targeting sequences of XPA-specific siRNAs (GENERAI BIOL) are: siXPA-1: 5’-GGAGACGAUUGUUCAUCAATT-3’; siXPA-2: 5’-CAGAGAUGCUGAUGAUAAATT-3’; siXPA-3: 5’-GGGUAGUCAAGAAGCAUUATT-3’.

### Western blotting, co-immunoprecipitation, and mass spectrometric analysis

Western blotting (WB) and co-immunoprecipitation (co-IP) were performed as previously described [22].

The Flag-tagged USP35 expression plasmids or the empty vectors were transfected into the HCT116 cell line. The USP35-associated proteins were co-immunoprecipitated with anti-Flag antibody. The proteins were then separated by SDS-PAGE and stained with Fast Silver Stain Kit (Beyotime Biotechnology). The band of interest was cut for mass spec. analysis according to previously mentioned protocol [22].

### Antibodies and reagents

The antibodies used in our experiments were listed in Table S2. 5-Fluorouracil (5-FU, APExBIO) and oxaliplatin (OXA, GlpBio) were stocked at a concentration of 3 and 10 mg/mL in water, respectively. MG132 and cycloheximide (Calbiochem) were stocked and used as previously described [22].

### Cell viability, apoptosis, and immunofluorescence

The cell viability was measured by clonogenic and Cell Counting Kit-8 (CCK-8, APExBIO) assays. For clonogenic assay, 2000 cells were seeded in the 6-well plates. Seven days after the seeding, cells were washed with PBS, fixed with 4% paraformaldehyde in PBS, and stained with Giemsa staining solution (Solarbio Life Science). For CCK-8 assay, 2000 cells were seeded in the 96-well plates for 48 h to examine the cell viability. Or else, 2000 cells were seeded in the 96-well plates for 24 h, and then the indicated drugs were added into the cells for 48-h treatments at different concentrations or for different time duration to examine the drug toxicity. The cell viability was examined by CCK-8 according to the manufacturer’s instruction.

The cell apoptosis was measured by Annexin V/Propidium Iodide (PI) staining followed by flow cytometry analysis or terminal dUTP nick-end labeling (TUNEL) assay as previously described [22].

The immunofluorescence (IF) staining and confocal microscopy were performed following the similar procedures as previously done [22].

### Proximity ligation assay (PLA)

PLA was performed using the Duolink® In Situ Red Starter Kit Mouse/Rabbit (Sigma-Aldrich). Briefly, DLD-1 and LoVo cells were grown on glass coverslips and fixed using 4% paraformaldehyde. The cells were washed with glycine-contained PBS and permeabilized with 0.1% Triton X-100-contained PBS for 20 min. The cells were blocked and incubated with anti-USP35 and anti-FUCA1 antibodies (Proteintech) diluted in blocking solution overnight at 4 °C. The pre-diluted anti-rabbit plus and anti-mouse minus probes were incubated at 37 °C for 1 h. Then, the cells were incubated with 1× ligase for 30 min and 1× polymerase for 100 min at 37 °C. Finally, coverslips were mounted on the slide with Duolink® In situ Mounting Medium with DAPI.

### Xenograft mouse model

Five-week-old male athymic nude mice were purchased from HFK Bioscience Company. In all, 5 × 10^5^ HT29 cells were subcutaneously inoculated into the flanks of the mouse (5 mice per group). A week after the inoculation, water, OXA (10 mg/kg in water), or OXA (5 mg/kg in water) plus 5-FU (50 mg/kg in water), was intraperitoneally injected in the mice weekly, and tumor size was measured every 3 days. The tumor volume (V) was calculated by the formula: *V* = 0.5 × length × width^2^. Mice were euthanized by cervical dislocation at the end of the experiment. The animals were bred in pathogen-free conditions and experimental procedures were approved by the Institutional Animal Care and Use Committee of Shandong University.

### Immunohistochemistry and scoring

30 human CRC tissues along with their adjacent non-cancerous tissues were obtained from the tumor tissue bank from the Affiliated Hospital of Qingdao University (Qingdao, China). Informed consent was obtained from the patients for this study. The immunohistochemistry (IHC) staining and the blind scoring were performed as previously described by two pathologists [20, 33].

### Statistical analysis

GraphPad Prism 8 software (La Jolla, CA, USA) was used for statistical analyses. The quantified data are presented as the mean ± SD of at least three independent experiments. Different groups were compared using unpaired, two-tailed, Student’s *t* test. Spearman’s correlation analysis was used for analyzing the correlation between the expression of USP35 and FUCA1 in the CRC samples. *p* < 0.05 was considered statistically significant.

## Results

### USP35 is overexpressed in CRC patients

**Fig. 1:**
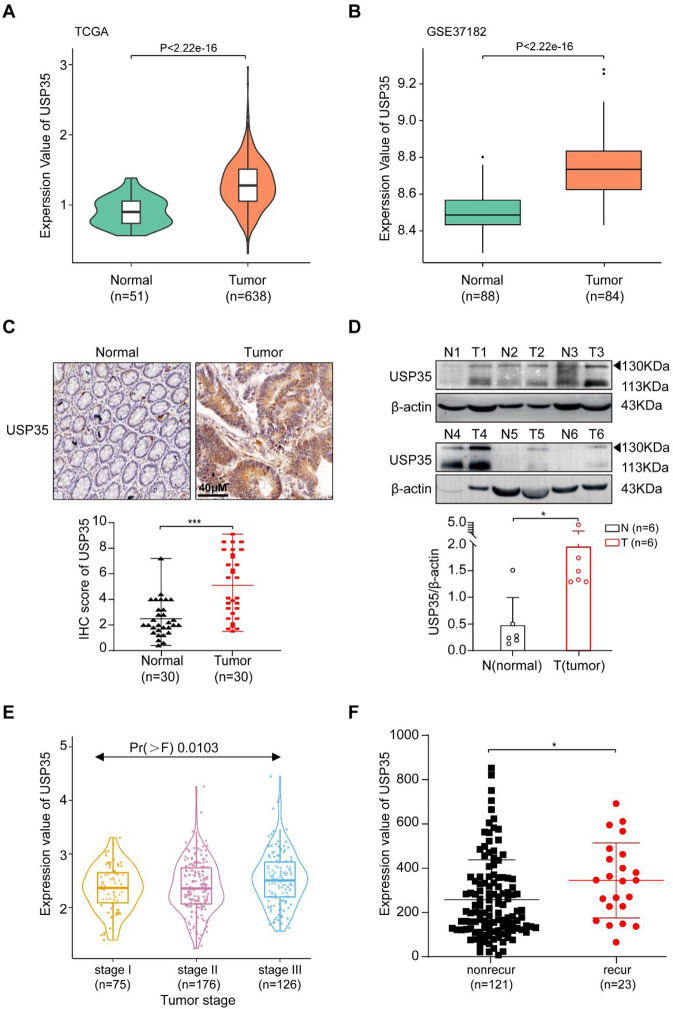
USP35 is overexpressed in CRC patients. **A**, **B** USP35 mRNA levels were higher in CRC patients according to the TCGA (**A**) and GEO (GSE37182) databases (**B**). **C** The representative immunohistochemistry staining of USP35 in CRC tissues and adjacent tissues (*n* = 30). Quantitative analysis was shown in the graphs. **D** The expression levels of USP35 in CRC tissues and adjacent tissues were detected by western blotting (*n* = 6). Quantitative analysis was shown in the graphs. **E** High expression of USP35 was associated with increased cancer stages in CRC patients based on TCGA database. **F** The expression of USP35 was higher in the patients with recurrent (recur) CRC compared the patients with nonrecurrent (nonrecur) CRC after receiving chemotherapy. The data were extracted from the TCGA database. All data are presented as mean ± SD. *p < 0.05, ***p < 0.001 based on the Student’s *t* test.

To explore the role of USP35 in CRC, we first checked its expression in CRC patients. According to the TCGA and GEO (GSE37182, GSE21815, and GSE71187) databases, USP35 mRNA levels were increased in CRC patients (Figs. 1A, B and S1). We further examined USP35 protein expression in our CRC patient samples through immunohistochemistry (IHC) staining and western blotting (WB). Consistently, the USP35 expression was higher by approximately two folds in the cancerous tissues compared to the non-cancerous tissues (Fig. 1C, D). Moreover, the TCGA data also suggested that high expression of USP35 was associated with increased cancer mutational in CRC patients and higher recurrence rate in CRC patients receiving postoperative chemotherapy (Fig. 1E, F). These data suggest that USP35 is elevated in CRC, and overexpression of USP35 is associated with CRC progression and recurrence, potentiating USP35 as a cancer target in CRC.

### USP35 promotes CRC cell proliferation and chemo-resistance

**Fig. 2:**
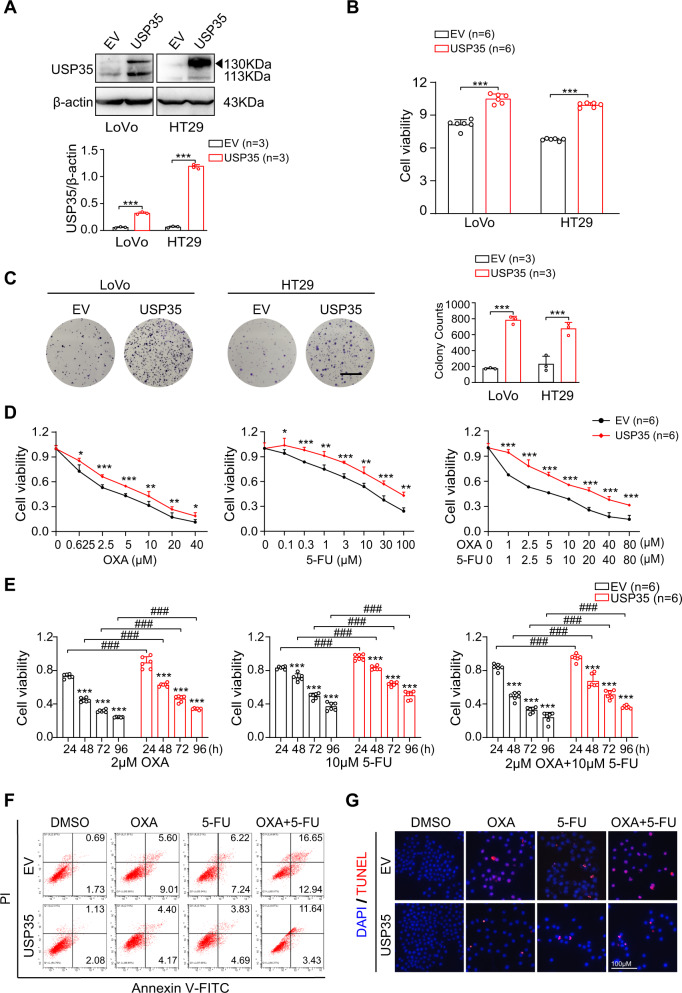
USP35 promotes CRC cell proliferation and chemo-resistance. **A** Establishment of USP35-overexpressed LoVo and HT29 cell lines. Quantitative analyses were shown in the graphs. **B** The cell viability of USP35-overexpressed LoVo and HT29 cells. Cells were implanted in 96-well plates for 48 h (*n* = 6) and the cell viability was assessed using CCK8 assay. **C** Representative images of clonogenic assay (*n* = 3). USP35 overexpression promoted cell proliferation. Quantitative analyses were shown in the graphs. The scale bars in represented 50 μm. **D** The USP35-overexpressed HT29 cells and the control cells were treated with different concentrations of oxaliplatin (OXA), 5-fluorouracil (5-FU), or combination of OXA and 5-FU for 48 h (*n* = 6). The cell viability was assessed using CCK8 assay. **E** The USP35-overexpressed HT29 cells and the control cells were treated with OXA (10 μM), 5-FU (10 μM), or combination of OXA (10 μM) and 5-FU (10 μM) for 24, 48, 72, or 96 h (*n* = 6). The cell viability was assessed using CCK8 assay. **F**, **G** The USP35-overexpressed HT29 cells and the control cells were treated with DMSO, OXA (10 μM), 5-FU (10 μM) or combination of OXA (10 μM) and 5-FU (10 μM) for 48 h (*n* = 3). Representative images indicated the apoptotic cells detected by flow cytometry analysis (**F**) and TUNEL staining (**G**). Data are presented as mean ± SD. *p < 0.05, **p < 0.01, ***p < 0.001, ^#^^##^*p* < 0.001 based on the Student’s *t* test.

To understand the impact of USP35 on CRC cell proliferation, we overexpressed USP35 in LoVo and HT29 cells, and depleted USP35 in DLD-1 and HCT116 cells (Figs. 2A and S2A). The cell proliferation was monitored by Cell Counting Kit-8 (CCK-8) and clonogenic assays. We found that augmented expression of USP35 promoted cell viability as well as clonogenic activity in LoVo and HT29 cells (Fig. 2B, C). USP35 deficiency, however, dampened the proliferation in DLD-1 and HCT116 cell lines, as indicated in Fig. S2B, C. These results show that USP35 is required for CRC cell proliferation.

Since USP35 expression was comparatively higher in CRC recurrent cases (Fig. 1F), we next investigated whether USP35 contributed to chemo-resistance in CRC cell lines. Oxaliplatin (OXA) and 5-fluorouracil (5-FU) are classical drugs in the CRC chemotherapy regimens, either as single or combination therapy [3]. We therefore examined whether USP35 affected CRC cell response to OXA, 5-FU, or the combined treatment of OXA and 5-FU. We first tested whether increased USP35 expression conferred resistance to OXA, 5-FU, or combination of OXA and 5-FU in HT29 cell lines. We treated the HT29 cells using single or double agents with different concentration or at different time points, and examined the cell viability through CCK-8 assay. We found that USP35 overexpression rendered HT29 cells more resistant to the cytotoxic agents, either on the condition of single or combined treatments (Fig. 2D, E). We then tested whether USP35 depletion sensitized cells to the chemotherapies using the similar methods, and found that USP35 ablation enhanced cytotoxic effects of those drugs on the DLD-1 cells (Fig. S2D, E). The impact of USP35 on cell apoptosis in response to OXA, 5-FU, or combination of OXA and 5-FU was further detected by Annexin V/Propidium Iodide (PI) staining flow cytometry analysis and terminal dUTP nick-end labeling (TUNEL) assay. Strikingly, USP35 overexpression markedly reduced cytotoxic agents-induced cell apoptosis (Fig. 2F, G), while USP35 knockdown led to increased drug-induced cell apoptosis (Fig. S3C, D). The quantified data of apoptotic cells were presented in Fig. S3A–D. In parallel, western blotting analysis showed that OXA, 5-FU, or combination of OXA and 5-FU treatment led to significant increases in the levels of the apoptosis markers, cleaved PARP1 and cleaved Caspase-3, while forced USP35 expression in HT29 cells reduced cytotoxic agents-induced increase of these proteins (Fig. S4A). On the contrary, USP35 silencing in DLD-1 cells dramatically boosted the increase of these apoptotic markers induced by these chemotherapeutic drugs, further supporting the chemo-resistant role of USP35 in CRC (Fig. S4B).

### USP35 deubiquitinates and stabilizes FUCA1

**Fig. 3:**
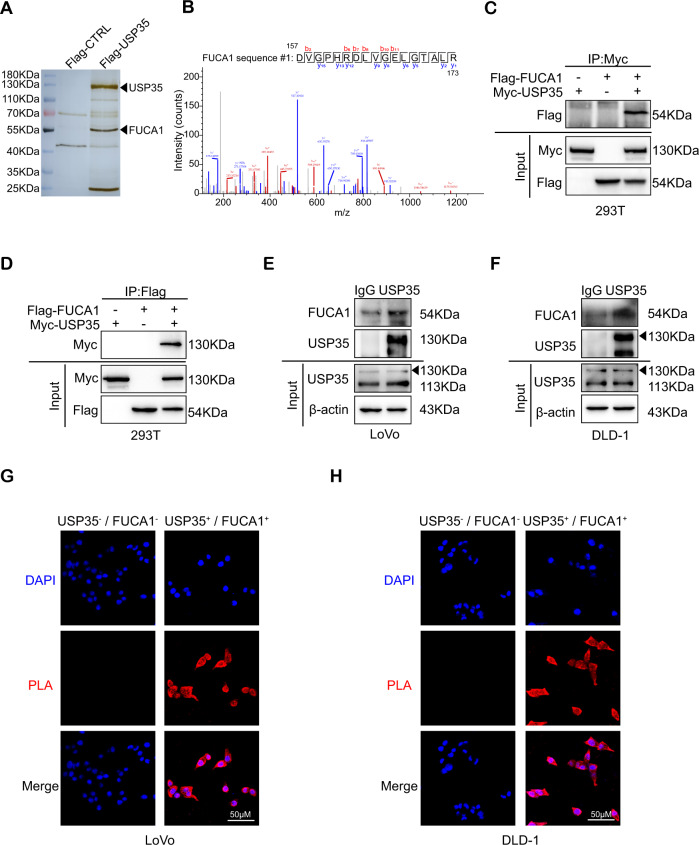
USP35 interacts with FUCA1. **A** Silver staining of the SDS-PAGE gel containing anti-Flag antibody immunoprecipitated proteins in Flag-USP35-overexpressed HCT116 cells and the control cells. **B** Representative map of mass spectrometry peaks of FUCA1 interacting with Flag-USP35. **C**, **D** Exogenous interaction between USP35 and FUCA1. HEK293T cells were co-transfected with Flag-FUCA1 and/or Myc-USP35 plasmids. Cell lysates were immunoprecipitated with indicated antibodies, followed by immunoblotting with anti-Flag antibody (**C**) or anti-Myc antibody (**D**). **E**, **F** Endogenous interaction between USP35 and FUCA1. Cell lysates from LoVo (**E**) and DLD-1 (**F**) were immunoprecipitated with anti-USP35 antibody or IgG antibody, followed by immunoblotting with anti-FUCA1 antibody. **G**, **H** Proximity ligation assay (PLA) showing USP35 and FUCA1 interaction in LoVo (**G**) and DLD-1 (**H**) cells. The results were the representative of three independent experiments.

To investigate the possible mechanism for USP35-mediated cell proliferation and drug resistance, we transfected Flag-tagged USP35 expression plasmid into the HCT116 cell line, immunoprecipitated the USP35-associated proteins with anti-Flag antibody, separated the proteins by SDS-PAGE, and analyzed the immunoprecipitated proteins by mass spectrometry (Fig. 3A). Mass spectrometry identified seven candidates (PRMT5, PPM1B, FUCA1, DNAJA1, IGF2BP1, DDX5, XRCC6) which may be associated with USP35 (Fig. S5A). Among these potential targets of USP35, we would like to specifically focus on α-L-fucosidase 1 (FUCA1) in our current study (Figs. 3B and S5B, C), considering the strong relevance of altered glycosylation in cancer development and therapeutics [24].

To validate our screened results, we first examined whether the physical interaction existed between USP35 and FUCA1. Co-immunoprecipitation (co-IP) assay was used for this purpose. HEK293T cells were transfected with Myc-tagged USP35 and/or Flag-tagged FUCA1, the USP35- or FUCA1-associated proteins were immunoprecipitated with anti-Myc or anti-Flag antibodies, and the lysates were subject to western blotting analysis. As illustrated in Fig. 3C, D, USP35 interacted with FUCA1 in a reciprocal fashion. The interaction between endogenous USP35 and FUCA1 was also detected in LoVo and DLD-1 cells (Fig. 3E, F). Moreover, we checked the interaction of endogenous USP35 and FUCA1 in CRC cells using proximity ligation assay (PLA). The specific positive signals (shown in red) were observed in the cells, further indicating the physical association between USP35 and FUCA1 (Fig. 3G, H). We also examined the localization of USP35 and FUCA1 by immunofluorescence (IF) staining followed by confocal microscopy, and found that USP35 and FUCA1 were expressed in both cytosol and nucleus (Fig. S5D, E).

**Fig. 4:**
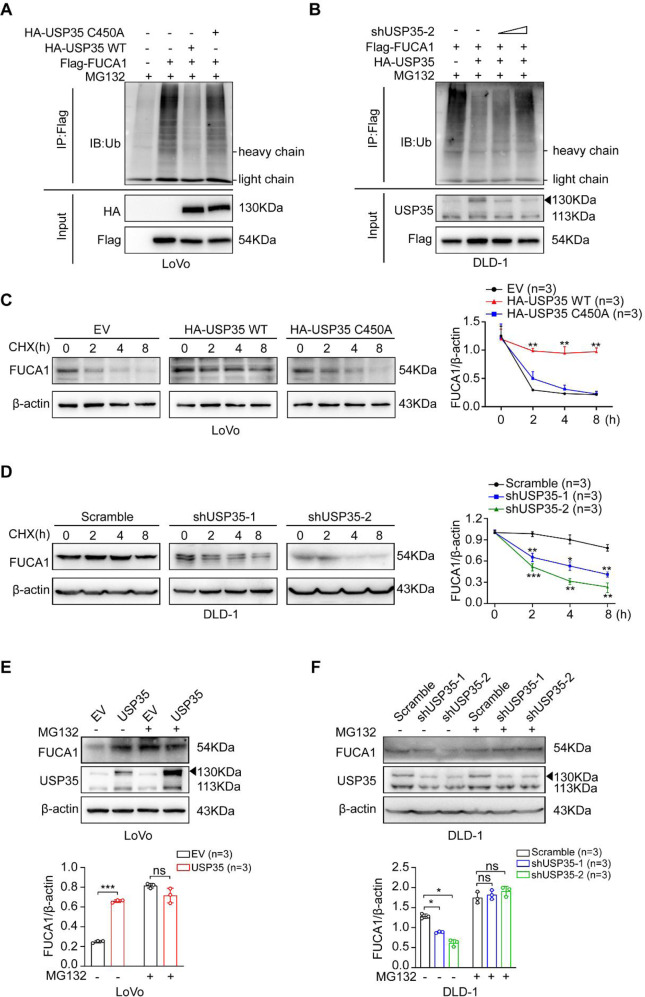
USP35 deubiquitinates and stabilizes FUCA1. **A**, **B** USP35 deubiquitinates FUCA1. **A** LoVo cells were transfected with Flag-FUCA1 alone or along with HA-tagged wild type USP35 (HA-USP35 WT) or the catalytically inactive USP35 (HA-USP35 C450A). Cell lysates were immunoprecipitated with anti-Flag antibody, followed by immunoblotting with anti-Ub antibody. MG132 was used to enrich the USP35 and FUCA1 proteins in the cells. **B** DLD-1 cells were transfected with Flag-FUCA1 alone or along with HA-USP35 WT and/or shUSP35-2 (0, 2 µg, or 4 µg). Cell lysates were immunoprecipitated with anti-Flag antibody, followed by immunoblotting with anti-Ub antibody. **C**, **D** USP35 increases the stability of FUCA1. LoVo cells expressing empty vector, HA-USP35 WT, or HA-USP35 C450A (**C**), and DLD-1 cells expressing USP35 specific scramble RNA, shUSP35-1, or shUSP35-2 (**D**) were treated with 50 μg/ml cycloheximide (CHX) for indicated time points. Then, the expression of FUCA1 was detected by western blotting. Quantitative analyses of CHX chase data are shown in the graphs. **E**, **F** USP35-mediated stabilization of FUCA1 is dependent on ubiquitin-proteasome system. USP35-overexpressed LoVo cells (USP35) and the control cells (EV) (**E**), and USP35-depleted DLD-1 cells (shUSP35-1 and shUSP35-2) and the control cells (Scramble) (**F**) were treated with or without 10 µM MG132 for 6 h. The expression of FUCA1 was detected by western blotting. Quantitative analyses were shown in the graphs. Data are presented as mean ± SD of three independent experiments. ns: not significant. *p < 0.05, **p < 0.01, ***p < 0.001 based on the Student’s *t* test.

Considering USP35 is a deubiquitinase, we then investigated whether FUCA1 was deubiquitinated by USP35. We transiently transfected HEK293T cells with Flag-tagged FUCA1, HA-tagged wild-type USP35 (USP35 WT), or HA-tagged catalytically inactive USP35 (USP35 C450A) [34] plasmids, immunoprecipitated FUCA1 associated proteins with anti-Flag antibody, and probed for ubiquitin (Ub) by western blotting. The proteasome inhibitor MG132 was used to enrich the USP35 and FUCA1 proteins in the cells. We found that only the catalytically active USP35 (USP35 WT), not the USP35 mutant (USP35 C450A), deubiquitinated FUCA1 (Fig. 4A). This result indicated that USP35 removed ubiquitins from FUCA1 and its enzymatic activity was required for this function. In addition, we silenced the USP35-overexpressed cells with different dose of shRNAs, and found that the ubiquitination of FUCA1 was increased with USP35 depletion in a dose dependent manner (Fig. 4B). This result further confirmed that FUCA1 was a deubiquitination target of USP35.

Given the most widespread outcome of protein ubiquitination is the alteration of protein stability [35], we next investigated whether USP35 affected FUCA1 stability. Cycloheximide (CHX) chase assay was used to measure the stability of FUCA1 with different USP35 expression levels. We found that overexpression of wild-type USP35 (USP35 WT), not the catalytically inactive mutant USP35 (USP35 C450A), prevented FUCA1 from degradation in LoVo cells, whereas knockdown of USP35 accelerated FUCA1 degradation in DLD-1 cells (Fig. 4C, D). Resultantly, enhanced USP35 expression increased FUCA1 at protein levels in CRC cells and HEK293T in a dose dependent manner (Fig. S6A, B), while dampened USP35 expression clearly decreased the protein levels of FUCA1 in DLD-1 and HCT116 cells (Fig. S6C). As expected, USP35 overexpression or silencing did not affect the mRNA levels of FUCA1 (Fig. S6D, E). Since proteasome is required in the ubiquitin-proteasome pathway for protein degradation, we further tested how altered expression of USP35 would affect the levels of FUCA1 in the presence of MG132. As a result, forced expression of USP35 in LoVo and HT29 cells failed to “wax” FUCA1, and depletion of USP35 in DLD-1 and HCT116 cells failed to “wane” FUCA1 as it usually did with addition of MG132 (Fig. 4E, F), indicating USP35 protected FUCA1 from proteasome-mediated degradation. Overall, these results demonstrate that USP35 deubiquitinates FUCA1 and increases FUCA1 stability by preventing ubiquitin-proteasome pathway-mediated degradation of FUCA1.

### FUCA1 mediates the effect of USP35 on cell proliferation and chemo-resistance in vitro

Considering the previously reported role of FUCA1 in cancer growth and chemo-resistance [27–31], we here investigated whether the influence of USP35 on cell proliferation and drug resistance was mediated or partially mediated by FUCA1 in our study.

**Fig. 5:**
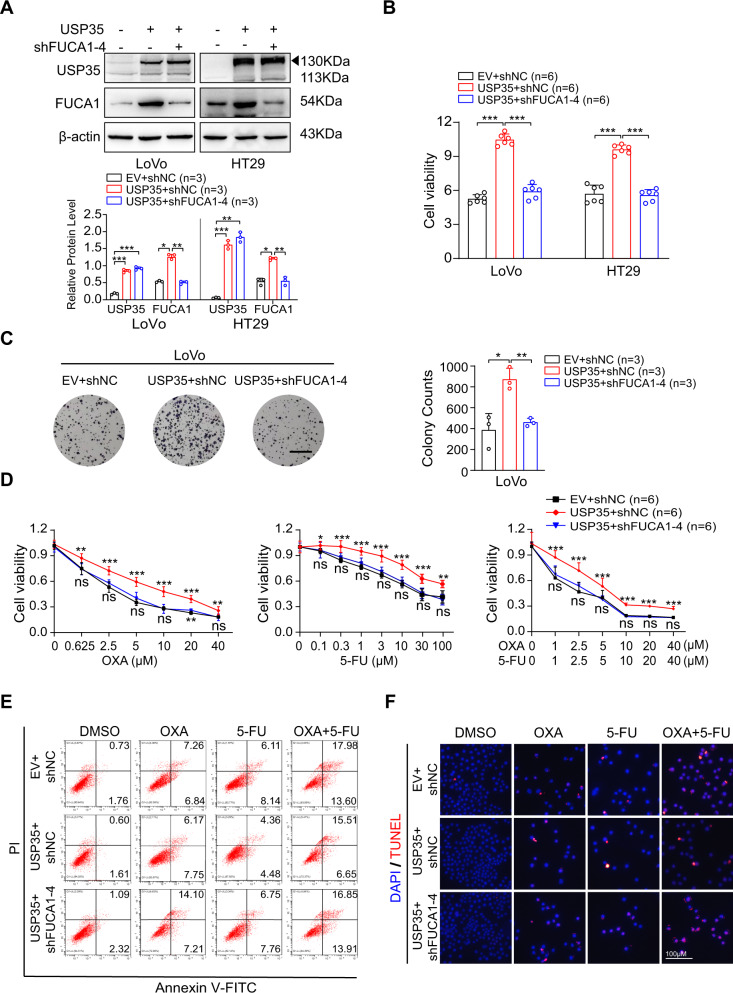
FUCA1 mediates the effect of USP35 on cell proliferation and chemo-resistance in vitro. **A** FUCA1-specific shRNA (shFUCA1-4) or control shRNA was introduced into the USP35-overexpressed HT29 and LoVo cells. The expression of FUCA1 and USP35 was detected by western blotting. Quantitative analysis was shown in the graphs. **B**, **C** CCK8 assay (**B**) and clonogenic assay (**C**) were used to detect the effect of FUCA1 depletion on USP35-induced cell proliferation. The scale bars in **C** represented 50 μM. **D** FUCA1 specific shRNA (shFUCA1-4) or control shRNA was introduced into the USP35-overexpressed HT29 cells. The cells were treated with different concentrations of OXA, 5-FU, or combination of OXA and 5-FU for 48 h (*n* = 6). The cell viability was assessed using CCK8 assay. **E**, **F** FUCA1-specific shRNA (shFUCA1-4) or control shRNA was introduced into the USP35-overexpressed HT29 cells. The cells were treated with DMSO, OXA (10 μM), 5-FU (10 μM), or combination of OXA (10 μM) and 5-FU (10 μM) for 48 h (*n* = 3). Representative images indicated the apoptotic cells detected by flow cytometry analysis (**E**) and TUNEL staining (**F**). Data are presented as mean ± SD. ns: not significant. *p < 0.05, **p < 0.01, ***p < 0.001 based on the Student’s *t* test.

We first knocked down FUCA1 in USP35-overexpressed LoVo and HT29 cells, using FUCA1-specific shRNA-4 with higher knockdown efficiency (Fig. S7A, B). As shown in Fig. 5A, FUCA1 expression was reduced to approximately the basal levels by FUCA1-specific shRNA-4 in the USP35-overexpressed LoVo and HT29 cells. Interestingly, FUCA1 ablation almost completely reversed proliferation-promoted effect of USP35 on LoVo and HT29 cells, as indicated by clonogenic and CCK-8 assays (Fig. 5B, C). Then, we stably overexpressed FUCA1 in USP35-depleted DLD-1 and HCT116 cell lines to restore FUCA1 expression to the near-basal levels (Fig. S8A). Consequently, augmented FUCA1 substantially ameliorated the growth-inhibitory effect of USP35 deficiency on these cells (Fig. S8B, C).

Next, we investigated whether USP35-triggered OXA and 5-FU resistance was associated with FUCA1 expression. Given previously, FUCA1 was identified as a direct transcriptional target of p53 and was required for cisplatin sensitivity [27], we determined to understand whether FUCA1 expression/function was dependent on p53 status/activation. Unlike wild-type p53 which is a classic tumor suppressor, p53 mutant has oncogenic potentials [36], therefore we examined whether p53-FUCA1 axis exists in wild-type p53 (HCT116 and LoVo) and p53 mutant (DLD-1 and HT29) CRC cell lines. We first examined whether FUCA1 expression was increased by drug-induced p53 activation. We found that in HCT116 and LoVo (wild-type p53) cells, FUCA1 and p53 expression were concurrently triggered by OXA or 5-FU treatments (Fig. S9C, D). However, upon chemotherapeutic insult, FUCA1 expression was not induced in parallel with p53 expression in p53 mutant CRC cells (Fig. S9A, B). This finding indicated that mutant p53 failed to regulate FUCA1 expression in CRC cells. We next investigated whether the function of FUCA1 was affected by p53 status. We found FUCA1 depletion alone did not affect cell apoptosis in either p53 wild-type or mutant CRC cells (Fig. S10). However, upon drug treatments (single OXA, single 5-FU, or combined OXA and 5-FU), FUCA1 deficiency led to different results dependently on the p53. While FUCA1 knockdown made cell much more resistant to the drug treatments in LoVo (wild-type p53) cells, FUCA1 depletion moderately sensitized the HT29 cells (mutated p53) to the similar treatment (Fig. S10C, D). This observation suggested that the effect of altered FUCA1 expression on chemotherapeutic response was dependent on p53 status. As p53 mutation was found in approximately 60% of the CRC patients [36], we specifically focused on exploring the role of USP35-FUCA1 axis in p53 mutant DLD-1 and HT29 cells.

Cells were subject to OXA, 5-FU, or combination treatments using similar conditions as in Fig. 3, and the drug toxicity was measured by CCK-8 assay. As a result, FUCA1 depletion fully re-sensitized the USP35-overexpressed HT29 cells to different chemotherapeutic treatments (Figs. 5D and S11B). However, boosted FUCA1 expression largely de-sensitized USP35-depleted cells to the similar treatments in DLD-1 cells (Fig. S11A, C). Moreover, drug-induced apoptosis in these cells was illustrated by Annexin V/PI staining flow cytometry analysis and TUNEL assay. We found that depleting FUCA1 in USP35-overexpressed cells led to an increase of apoptotic cells to a rate as the control (EV + shNC) cells (Figs. 5E, F and S11D, E), while re-expressing FUCA1 in USP35-deficient cell decreased apoptotic cell population to the similar level as control (Scramble + Vector) cells in response to cytotoxic insults (Fig. S12A, B). Together, these data suggest that FUCA1 is an essential player that mediates USP35-associated cell proliferation and OXA/5-FU resistance in CRC cells.

### FUCA1 mediates the impact of USP35 on tumor growth and chemo-resistance in vivo

**Fig. 6:**
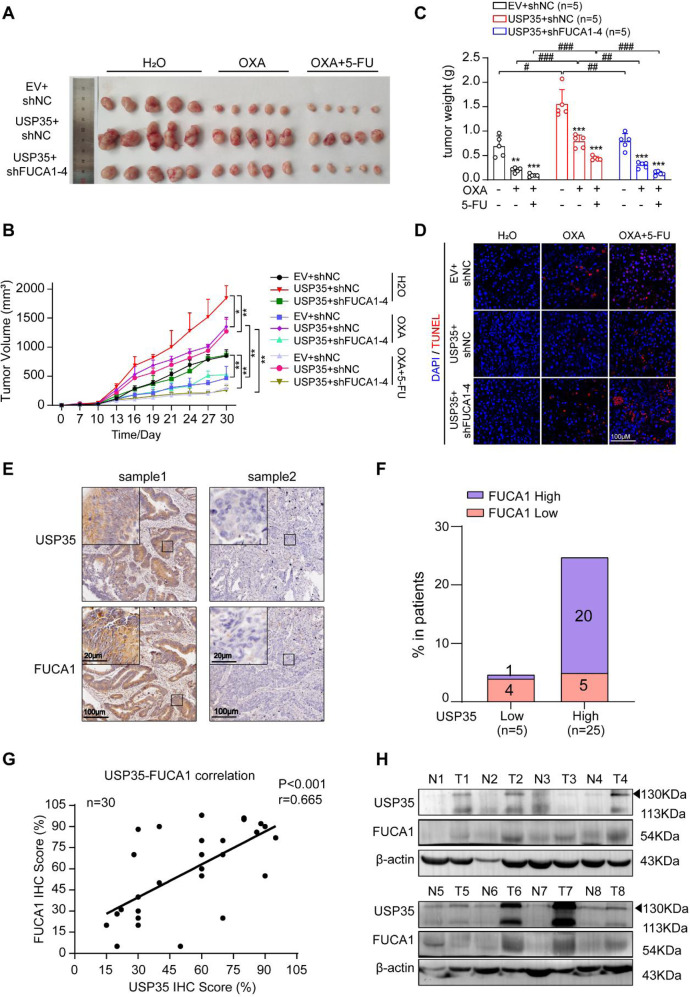
FUCA1 mediates the impact of USP35 on tumor growth and drug resistance in vivo. **B** Tumor growth curve of HT29 cells with or without altered expression of USP35 or FUCA1. Cells were subcutaneously injected into the nude mice (*n* = 5). A week after the inoculation, water, OXA (10 mg/kg), or OXA (5 mg/kg) plus 5-FU (50 mg/kg), was intraperitoneally injected in the mice weekly. Tumors were measured at the indicated time points. **A** Representative images of the dissected tumors were shown. A ruler is used to demonstrate the size of the tumors. **C** Quantification of tumor weights at the end point. **D** Cell apoptosis was detected using TUNEL assay of the tumor sections. **E** Representative immunohistochemistry (IHC) stainings of USP35 and FUCA1 in the human CRC tissues. **F** Box plot showing the relative FUCA1 levels in USP35-low and USP35-high patients (*n* = 30). Median USP35 or FUCA1 expression was defined as the cut-off point. **G** The correlation of the expression of USP35 and FUCA1 in the human CRC tissues by IHC staining (*n* = 30). **H** The expression levels of USP35 and FUCA1 in the human CRC tissues and adjacent tissues were detected by western blotting (*n* = 8). The data are shown as the mean ± SD. *p < 0.05, **p < 0.01, ***p < 0.001, ^#^*p* < 0.05, ^##^*p* < 0.01, ^###^*p* < 0.001 based on the Student’s *t* test.

To explore the role of USP35-FUCA1 axis in vivo, we subcutaneously inoculated the control (EV + shNC), USP35-overexpressed (USP35 + shNC), and USP35 overexpressed FUCA1-depleted (USP35 + shFUCA1-4) HT29 cells in the athymic nude mice, intraperitoneally injected water (H_2_O), single drug (OXA as a representative), or combined drugs (OXA + 5-FU) weekly into the mice, and routinely monitored the tumor growth. We observed that USP35 overexpression markedly promoted tumor growth and drug resistance, but depleting FUCA1 in USP35-overexpressed cells reversed the tumor growth to the basal levels, and re-sensitized the tumors to the chemo-treatments, as indicated by representative images, tumor weight and growth curve (Fig. 6A–C). Moreover, we performed immunohistochemical (IHC) staining of cleaved Caspase-3 and TUNEL assay in the xenograft samples. In parallel with the in vitro data, USP35 overexpression weakened drug-induced cell apoptosis while knockdown of FUCA1 in USP35-overexpressed cells restored drug-induced cell apoptosis, suggesting that USP35 contributes to OXA/5-FU resistance through stabilizing FUCA1 in vivo (Fig. 6D and S13A–C).

Next, we examined the USP35-FUCA1 axis in the human CRC tissues. A total of 30 human CRC samples were included for IHC staining and analysis. As shown in the representative images, USP35 and FUCA1 shared the similar expression pattern (Fig. 6E). For example, in some samples, USP35 and FUCA1 were both intensely expressed in the CRC epithelium and lightly stained in the CRC stroma (Fig. 6E, sample 1). In the samples with low USP35 expression, FUCA1 expression was usually barely detectable (Fig. 6E, sample 2). According to the assessment by the pathologists, among 25 USP35-highly expressed CRC samples, 20 samples expressed elevated FUCA1, and among 5 USP35-lowly expressed CRC samples, 4 samples exhibited reduced FUCA1 expression (Fig. 6F). The strong correlation between USP35 and FUCA1 expression levels in the CRC tissues was further verified by Spearman’s correlation analysis (*p* < 0.001 and *r* = 0.665, Fig. 6G). We also examined the expression of USP35 and FUCA1 in human CRC tissues as well as adjacent normal tissues by western blotting, and found that FUCA1 expression was largely in accordant with USP35 expression (Fig. 6H). The quantified western blotting data and correlation analysis were shown in Fig. S13D, E. Therefore, these results suggest that USP35 expression correlates well with FUCA1 expression in CRC patient samples, and USP35-FUCA1 axis contributes to CRC tumor growth and chemo-resistance in vivo.

### USP35-FUCA1 axis up-regulates nucleotide excision repair in CRC

Two studies demonstrated that USP35 deficiency sensitized cancer cells to cisplatin (the first platinum anti-cancer drug) treatment via de-stabilizing anti-apoptotic factor BIRC3 or activating cGAS-STING-TBK1-mediated expression of type I interferons [20, 23]. However, another study showed that FUCA1, as a direct transcriptional target of p53, was required for cisplatin-induced cell apoptosis [27]. Given that the latter study was in discrepant with our findings, which might be due to the different experimental settings, we here investigated the possible mechanism of USP35-FUCA1-mediated oxaliplatin (the third-generation platinum drug) resistance in CRC.

The Pt-based chemotherapies work by introducing DNA-platinum adducts, which is mainly corrected by nucleotide excision repair (NER) [37, 38]. Increased NER proficiency is one of the reasons accounting for on-target resistance of Pt-based therapy [37, 39]. We therefore examined the expression levels of some major components (e.g., XPC, XPA, ERCC1) of the NER system [40]. We found that in USP35-overexpressed LoVo and HT29 cells, the expression levels of XPC, XPA and ERCC1 were markedly increased (Figs. S14A, B and S15A, B). Accordingly, the XPC, XPA and ERCC1 levels were decreased by USP35 knockdown in a dose-dependent manner in DLD-1 and HCT116 cells (Figs. S14C, D and S15C, D). Moreover, ablating FUCA1 in HT29 cells dramatically reduced the expression of XPC, XPA and ERCC1, whereas enhancement of FUCA1 largely restored the level of these NER components (Figs. S14E, F and S15, F). In addition, to investigate the involvement of the NER pathway in USP35-mediated chemo-resistance, we silenced XPA in USP35-depleted FUCA1-overexpressed DLD1 cells using XPA-specific siRNA and examined cell apoptosis in response to chemotherapeutics by flow cytometry analysis. Strikingly, we found that XPA silencing partially rescue the phenotype, reinforcing the possibility that USP35-FUCA1 axis controls chemo-resistance by regulating the expression of NER components (Fig. S16A, B). However, the detailed mechanism how USP35-FUCA1 regulates the NER warrants further investigation.

Hence, our current results indicate that USP35-FUCA1 axis up-regulates NER in CRC cells, which may be a possible mechanism for USP35-FUCA1-mediated oxaliplatin resistance in CRC.

## Discussion

In this study, we for the first time investigated the role of USP35 in colorectal cancer. We have demonstrated that USP35 controls tumor growth and chemotherapeutic vulnerability in CRC by mediating the stability of FUCA1, indicating that USP35 is an intriguing target in CRC. This study adds another evidence for USP35-targeted therapy in cancer. Previous studies have demonstrated the therapeutic potential of USP35 in some types of cancers, such as lung cancer [20, 21], ovarian cancer [23], and breast cancer [41]. Of note, our previous studies have shown that USP35 ablation contributes to ER stress and cisplatin vulnerability in non-small cell lung cancer (NSCLC) [20, 42]. Although the variety of cancer types/subtypes, the complexity of cancer origins, and the heterogeneous nature of cancers together poses a big challenge to cancer treatments, understanding the molecular mechanisms of cancer development and drug resistance gives rise to smart combination therapies that ultimately become powerful anti-cancer arsenals [43]. Given that current studies have connected USP35 to therapeutic sensitivity in a few types of cancers, it’s worth investigating whether USP35 is a universal regulator of chemo-sensitivity in other types of cancers. These studies will assist in our understanding of molecular subtypes of cancer and provide further insight into USP35-targeted combination therapies.

Despite the limited studies exploring the functions of USP35 in cancers, recent evidence has suggested that USP35 is a potential cancer target that regulates tumorigenesis, cell death, and cancer immunology [19–23, 32]. Interestingly, out of these six studies, three studies investigated the role of USP35 in cell death, including RRBP1-mediated ERS-triggered apoptosis [22], BIRC3-mediated cisplatin-triggered apoptosis [20], and ferroportin-mediated iron overload-triggered ferroptosis [21]. Our study has further demonstrated that USP35 is a cell death-related deubiquitinase that prevents oxaliplatin- and 5-fluorouracil-induced apoptosis in CRC. Overall, the mechanism of cell death is highly diversified, which can be categorized into programmed apoptosis, programmed non-apoptotic cell death (e.g., ferroptosis, pyroptosis, mitoptosis, etc.), and necrosis [44]. While the current studies give us a glimpse of how USP35 participates in cell death, there is still a lot to explore to determine whether USP35 is indeed a master regulator that can affect cell death in multiple ways. Resisting cell death is one of the hallmarks of cancer and targeting cell death pathway (e.g., Bcl-2) has achieved encouraging results in clinics [45, 46]. Additionally, many apoptosis-targeted therapies are being actively tested in clinical trials [47]. Given the druggable nature of DUBs [12, 17], this study provides pre-clinical evidence that USP35 is an apoptotic-associated DUB that may be targeted and applied in the CRC therapeutics.

Apart from the cell death-associated role of USP35, two other aspects of USP35 seem to be very intriguing to us as well—its participation in mitosis and cancer immunology. A study led by Park et al. showed that USP35 was required for mitotic progression, and Aurora B was a deubiquitination target of USP35 [34]. The mitotic role of USP35 places it in an appealing spot for targeting, considering that mitosis is a promising anti-cancer target [48, 49]. Their study also raises a question whether targeting USP35 will modulate Taxol response in cancers. Taxol is a clinically used microtubule-targeting agent (MTA) that exerts the cytotoxic effect by inducing chromosome mis-segregation [50]. It is possible that USP35 inhibition may synergize with Taxol to induce apoptosis or cell arrest, based on the result that USP35 deficiency itself already leads to multiple mitotic errors, and this outcome could be further exacerbated by Taxol treatment [34]. As for the involvement of USP35 in cancer immunology, two studies indicated that USP35 overexpression was associated with immune-suppressive tumor microenvironment (TME), especially the decrease in CD8^+^ T cell infiltration [23, 51]. In the study of Zhang et al., the authors further illustrated that USP35 repressed cGAS-STING-interferon signaling, which could be the reason for reduced CD8^+^ T cell infiltration in ovarian cancer [23]. Although immuno-oncology is an old concept, it really ushered into a new era in cancer care with series of new drugs under development and investigation [52, 53]. Compared with traditional cancer treatments (e.g., chemotherapy and radiotherapy), immunotherapy has considerable advantages, such as high accuracy, thoroughness, less toxicity, etc. [54]. However, immunotherapy is also complicated, due to the largely differed response rate, which can be affected by tissue specificities, TME, and individual difference [53, 54]. Thus, understanding the molecular drivers for immune-suppressive TME will improve individualized treatment in cancer immunotherapy. The previous studies have connected USP35 to immune microenvironment to some levels, and future studies may shed more light on whether and how targeting USP35 will activate the immune system against cancers in different models.

Based on current findings of FUCA1, the role of FUCA1 in cancers still remains controversial [55]. On one hand, early studies have shown that FUCA1, as a direct target of p53, is capable of triggering cell death by overexpression, and required for cytotoxicity effect of cisplatin and etoposide [27, 28]. Additionally, overexpression of FUCA1 attenuates thyroid cancer cell motility, and FUCA1 stably depleted breast cancer cells develop increased proliferative and metastatic capabilities [29, 56]. On the other hand, studies have also demonstrated that FUCA1 is required for glioma growth in vitro and in vivo, and transient inhibition of FUCA1 leads to G1-S arrest in breast cancer cells [56, 57]. Moreover, FUCA1 has also been associated with suppressive TME in that FUCA1 depletion reduces tumor associated macrophage (TAM) recruitment to the tumor site in glioma [57]. Hence, our finding that FUCA1 mediates the function of USP35 on cell proliferation and chemo-resistance in CRC, which seems to contradict the result of a previous study claiming FUCA1 is required for cisplatin-induced cell apoptosis [27], is not utterly surprising. We believe the discrepancy may be due to the different cancer type, genetic background, or experimental settings. Interestingly, previous studies have connected FUCA1 deficiency to autophagic cell death in cancers, given the lysosomal enzyme nature of FUCA1 [56, 57]. It is therefore possible that dysregulated autophagy being one of the reasons why FUCA1 plays an important role downstream of USP35 in controlling cell proliferation and therapeutic vulnerability in CRC. Experiments detecting the change of autophagic flux and autophagic cell death will be of great help in further understanding the roles and mechanisms of USP35-FUCA1 axis in CRC.

In searching for the potential mechanism of how USP35–FUCA1 axis contributes to oxaliplatin resistance in CRC, we examined the expression of the main DNA repair pathway for platinum-induced DNA damage response (DDR)—the nucleotide excision repair (NER). We found that NER components (e.g., XPC, XPA and ERCC1) was up-regulated by the USP35-FUCA1 axis and we considered it an underlying mechanism for USP35-FUCA1 axis-mediated oxaliplatin resistance in CRC. Nevertheless, whether USP35-FUCA1 axis promotes the expression of NER components at transcriptional levels, post-transcriptional modifications, translational levels, or post-translational modifications, warrants further investigation. Despite the fact that targeted therapy and immunotherapy have considerably advanced in the past few years, platinum (Pt) compounds still remain one of the most frequently used anti-neoplastic drugs in the clinic [39]. Pt compounds are often used in combination with other chemotherapeutic agents to overcome drug resistance and reduce toxicity in clinical settings [38]. Additionally, novel combinations of Pt-based therapies that mediate immune checkpoint response, DNA damage repair system, reactive Pt accumulation, synergistic apoptosis, etc., are gaining increased interest over the years, and are being actively tested in pre-clinical studies [38, 39]. This study offers a novel target as well as a perspective for Pt-based combination therapy.

5-Fluorouracil is an anti-metabolic drug that inhibits thymidylate synthase (TS) activity and triggers DNA or RNA damage, and is commonly used for the treatments of many solid tumors [58]. Like all other conventional anti-cancer drugs, the clinical application of 5-FU is limited by the development of chemo-resistance [59]. The current known mechanisms of 5-FU resistance include altered metabolic enzymes, presence of cancer stem cells (CSCs) or emergence of CSCs-like properties, angiogenesis, increased DNA damage repair (e.g., base excision repair [BER], mismatch repair [MMR], and homologous recombination [HR]) [59]. Accordingly, several strategies have been proposed to overcome 5-fluorouracil resistance, such as combination therapy, improved drug delivery, use of resistance reversal agents, and inhibition of DNA damage repair [59]. Although our study has demonstrated that USP35–FUCA1 axis confers resistance to 5-FU in CRC, we did not go further to explore the underlying mechanism. This area of study acquires a thorough investigation in the future considering the diverse mechanisms of 5-FU resistance.

In summary, this study has elucidated the role of USP35 in CRC cell proliferation and drug resistance. We have also demonstrated that FUCA1 is an important mediator for USP35 functions in CRC. Given the enzymatic activity of USP35 and FUCA1, we provide an outlook that targeting USP35–FUCA1 axis could be a plausible strategy for restricting tumor growth and chemo-resistance in CRC. The role of USP35 in CRC will be further investigated in different animal models. Moreover, USP35 will be pharmacologically targeted to examine the druggability, as well as the biological significance of USP35 in CRC.

## Supplementary information

Supplementary Information File

## Data Availability

The USP35 gene expression data in human CRC tissues and the non-cancerous tissues were derived from the TCGA and GEO (GSE37182, GSE21815, and GSE71187) databases. The USP35 expression levels correlated with tumor stage as well as the recurrence rate in CRC patients receiving postoperative chemotherapy were derived from the TCGA database. All the data supporting the findings of this study are available from the corresponding author on reasonable request.
