# Supplementary material for: USP35 promotes cell proliferation and chemotherapeutic resistance through stabilizing FUCA1 in colorectal cancer
Source: Oncogenesis. 2023 Mar 3;12(1):12. doi: 10.1038/s41389-023-00458-2 (PMC9981583; doi:10.1038/s41389-023-00458-2)
Supplement: Supplementary file 1 — Supplementary Information File [file 41389_2023_458_MOESM1_ESM.docx]

**Supplementary Information File for**

**USP35 promotes cell proliferation and chemotherapeutic resistance through stabilizing FUCA1 in colorectal cancer**

Yi Xiao^1,2^, Xiaoyu Jiang^1^, Ke Yin^3^, Tianshu Miao^1^, Hanlin Lu^1,4^, Wenqing Wang^1^, Lijuan Ma^1^, Yinghui Zhao^5^, Chunyan Liu^6^, Yun Qiao ^7^*, Pengju Zhang^1^*

**Authors’ affiliation:**

*1, Key Laboratory Experimental Teratology of the Ministry of Education, Department of Biochemistry and Molecular Biology, School of Basic Medical Sciences, Cheeloo College of Medicine, Shandong University, Jinan, Shandong, 250012, China.*

*2, Eppley Institute for Research in Cancer and Allied Diseases, Fred & Pamela Buffett Cancer Center, University of Nebraska Medical Center, Omaha, NE, 68198, USA.*

*3, Department of Pathology, Shandong Provincial Hospital, Shandong University, Jinan, Shandong, 250021, China*

*4, Department of Cardiology, Qilu Hospital of Shandong University, Jinan, Shandong, 250012,China.*

*5, Department of Clinical Laboratory, The Second Hospital of Shandong University, No. 247 Beiyuan Street, Jinan, Shandong, 250033, China.*

*6, Department of Integrated Traditional Chinese and Western Medicine, Medical College of Qingdao University, Qingdao, Shandong, 266071, China.*

*7,* *Department of Traditional Chinese Medicine, Qilu Hospital of Shandong University, Jinan, Shandong, 250012, China.*

These authors contributed equally: Yi Xiao, Xiaoyu Jiang

*Corresponding author. Email: [zhpj@sdu.edu.cn](mailto:zhpj@sdu.edu.cn)

qiaoyun@qiluhospital.com

**This Supplementary Information File includes:**

Supplemental Figure 1-16

Supplemental Table 1-2

**Supplementary Figure S1.**


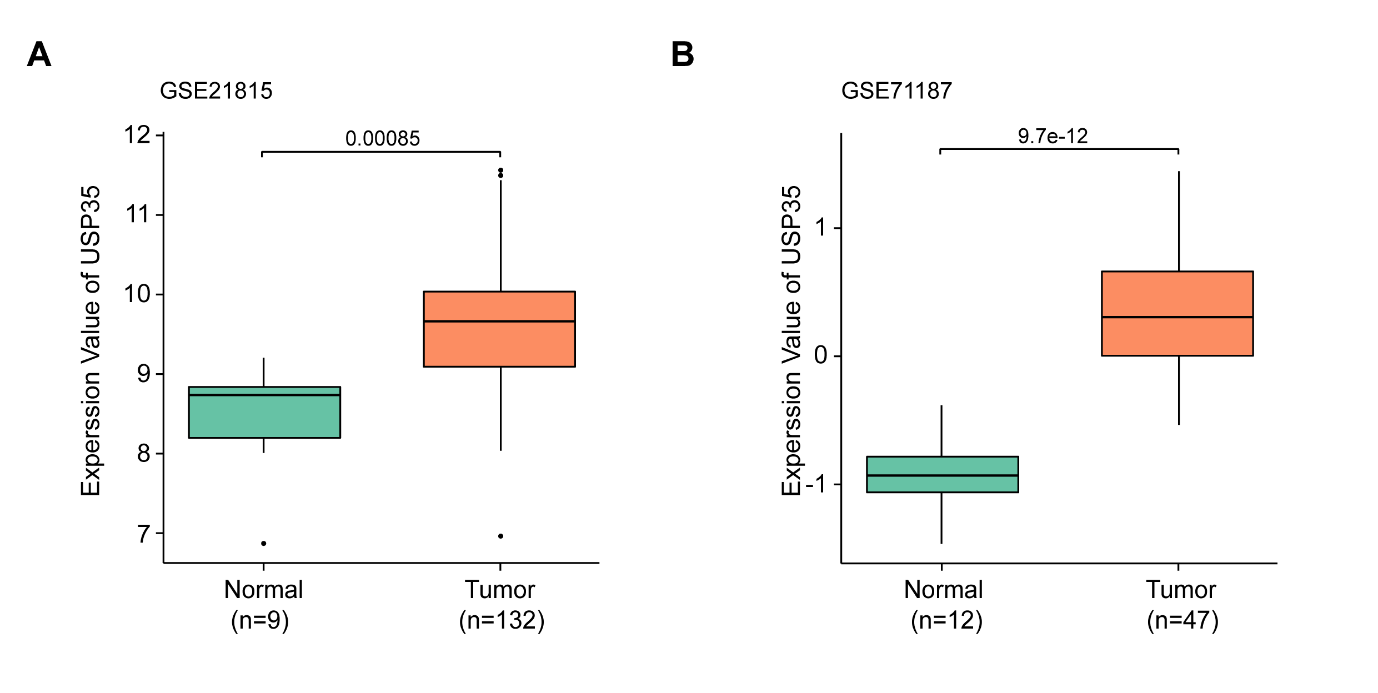


**Fig. S1. USP35 is overexpressed in CRC patients.** (A, B) The expression of USP35 at mRNA levels was higher in CRC patients according to the GEO databases: GSE21815 (A) and GSE711879 (B).

**Supplementary Figure S2.**

**
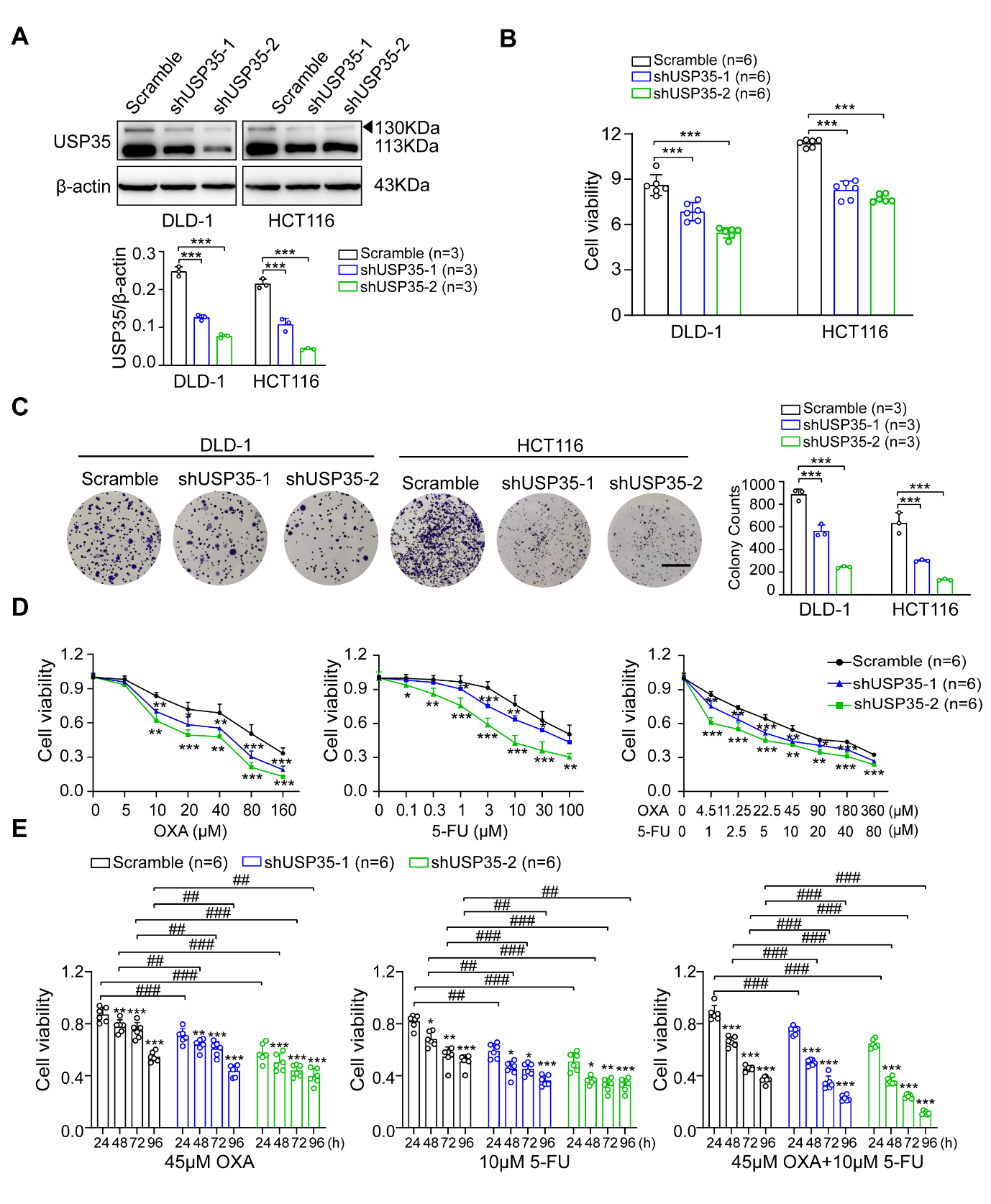
**

**Fig. S2. USP35 promotes CRC cell proliferation and chemo-resistance.** (A) USP35-depleted DLD-1 and HCT116 cell lines. Quantitative analyses were shown in the graphs. (B) The cell viability of USP35-depleted DLD-1 and HCT116 cells. Cells were implanted in 96-well plates for 48 hours (n = 6) and the cell viability was assessed using CCK8 assay. (C) Representative images of clonogenic assay (n = 3). USP35 knockdown hampered cell proliferation. Quantitative analyses were shown in the graphs. The scale bars in represented 50 μm. (D) The USP35-depleted DLD-1 cells and the control cells were treated with different concentrations of the OXA, 5-FU or combination of OXA and 5-FU for 48 hours (n = 6). The cell viability was assessed using CCK8 assay. (E) The USP35-depleted DLD-1 cells and the control cells were treated with OXA (45 μM), 5-FU (10 μM) or combination of OXA (45 μM) and 5-FU (10 μM) for indicated time points (n = 6). The cell viability was assessed using CCK8 assay. Data were presented as mean ± SD. *: p < 0.05, **: p < 0.01, ***: p < 0.001, ^##^: p < 0.01, ^###^: p < 0.001 based on the Student’s *t*-test.

**Supplementary Figure S3.**


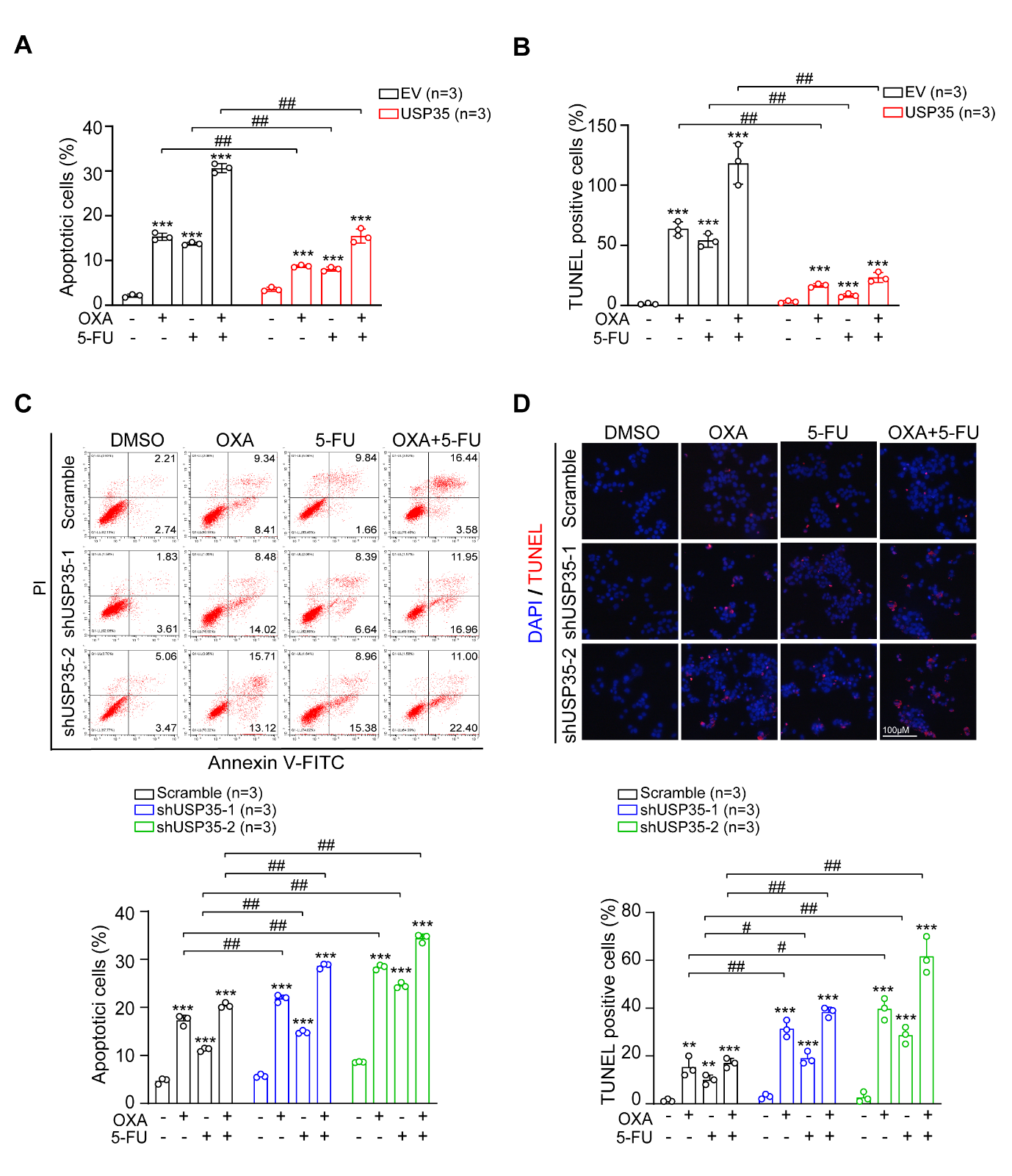


**Fig. S3. USP35 promotes chemo-resistance in CRC cells.** (A, B) Quantitative analyses (n = 3) of apoptotic rate of the USP35-overexpressed and the control cells HT29 cells by flow cytometry analysis (A) and TUNEL staining (B). (C, D) The USP35-depleted DLD-1 cells and the control cells were treated with DMSO, OXA (45 μM), 5-FU (10 μM) or combination of OXA (45 μM) and 5-FU (10 μM) for 48 hours respectively (n = 3). Representative images showed the apoptotic cells detected by flow cytometry analysis (C) and TUNEL staining (D). Quantitative analyses were shown in the graphs. Data were presented as mean ± SD. **: p<0.01, ***: p<0.001, ^#^: p < 0.05, ^##^: p < 0.01, based on the Student’s *t*-test.

**Supplementary Figure S4.**

**
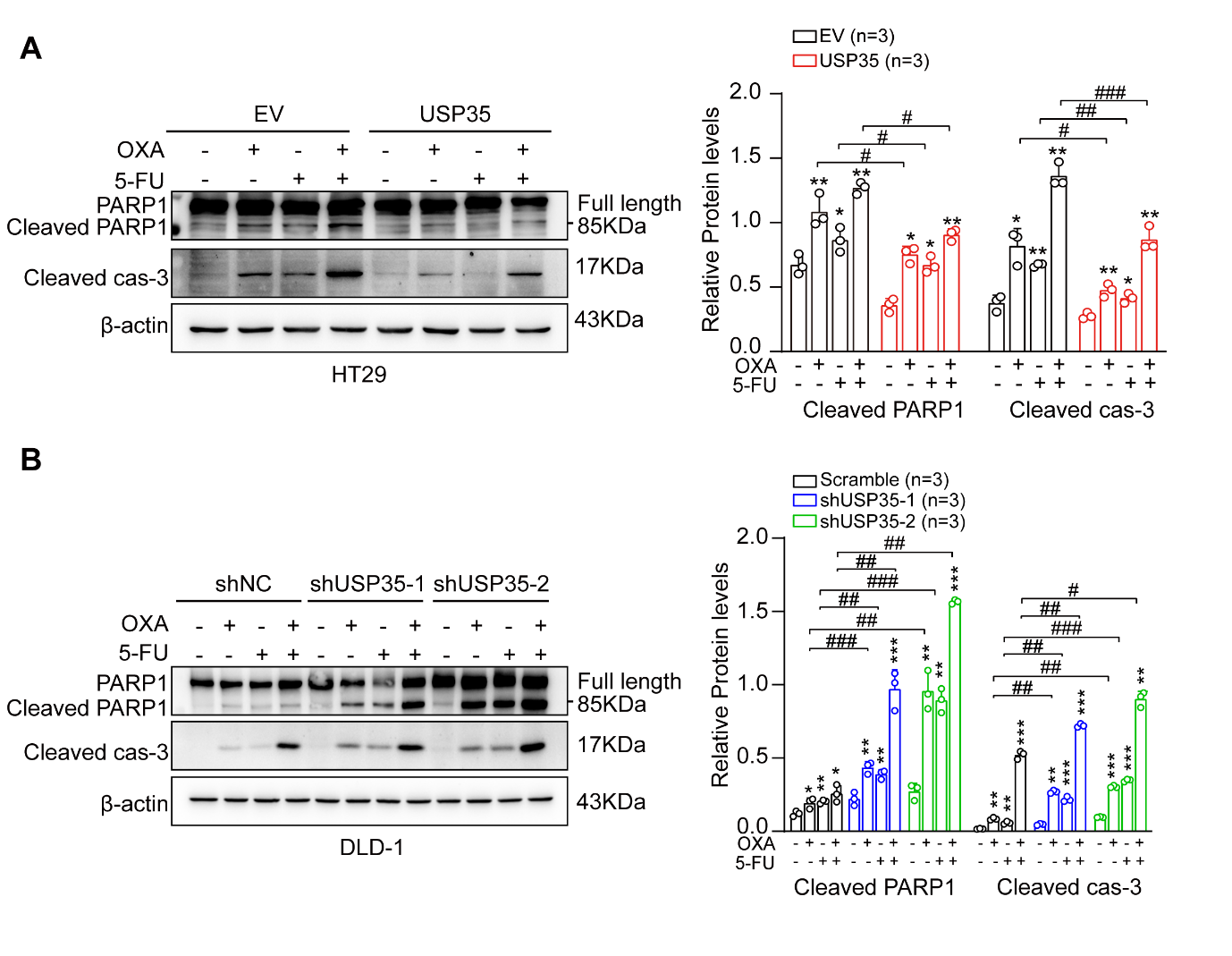
**

**Fig. S4. USP35 promotes chemo-resistance in CRC cells.** (A, B) The protein levels of the apoptotic markers, cleaved PARP1 and cleaved Caspase-3 (cas-3), in USP35 overexpressed HT29 cells (A) or USP35 silenced DLD-1 cells (B) treated with DMSO, OXA, 5-FU or combination of OXA and 5-FU for 48 hours respectively were detected by western blotting. Quantitative analyses were shown in the graphs (n=3). All data are presented by mean ± SD. *: P<0.05, **: P<0.01, ***: P<0.001; ^#^: P<0.05, ^##^: P<0.01, ^###^: P<0.001 based on the Student’s test.

**Supplementary Figure S5.**

**
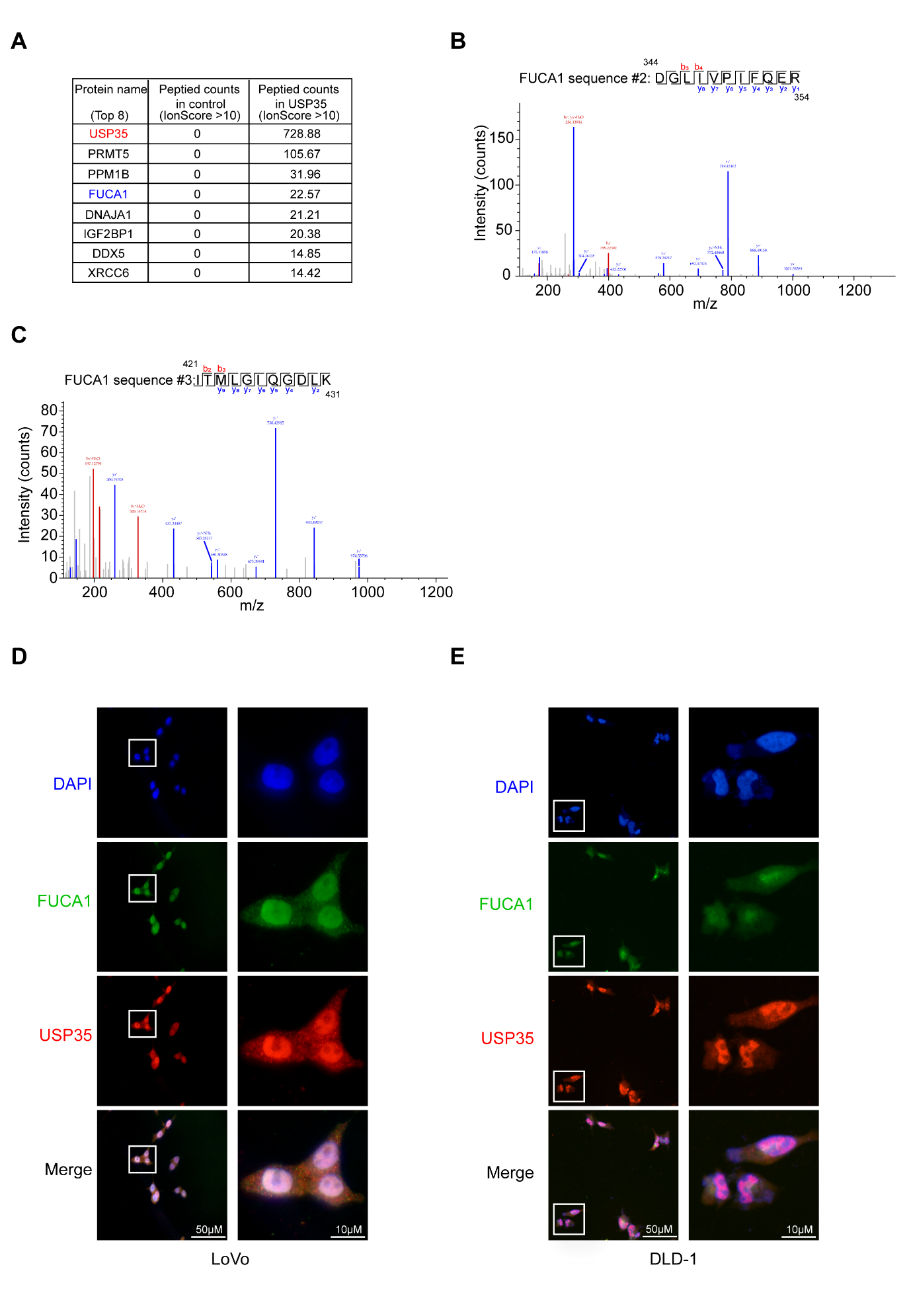
**

**Fig. S5. USP35 interacts with FUCA1.** (A) The top 7 abundant proteins (IonScore > 15) interacting with USP35 identified by mass spectrometry. (B, C) Maps of mass spectrometry peaks for FUCA1 interacting with USP35. (D, E) Immunofluorescence staining and confocal microscopy showing USP35 and FUCA1 localization in LoVo (D) and DLD-1 (E) cells. Merged images indicated the overlapped area of USP35 and FUCA1 localization. The results were the representative of three independent experiments.

**Supplementary Figure S6.**

**
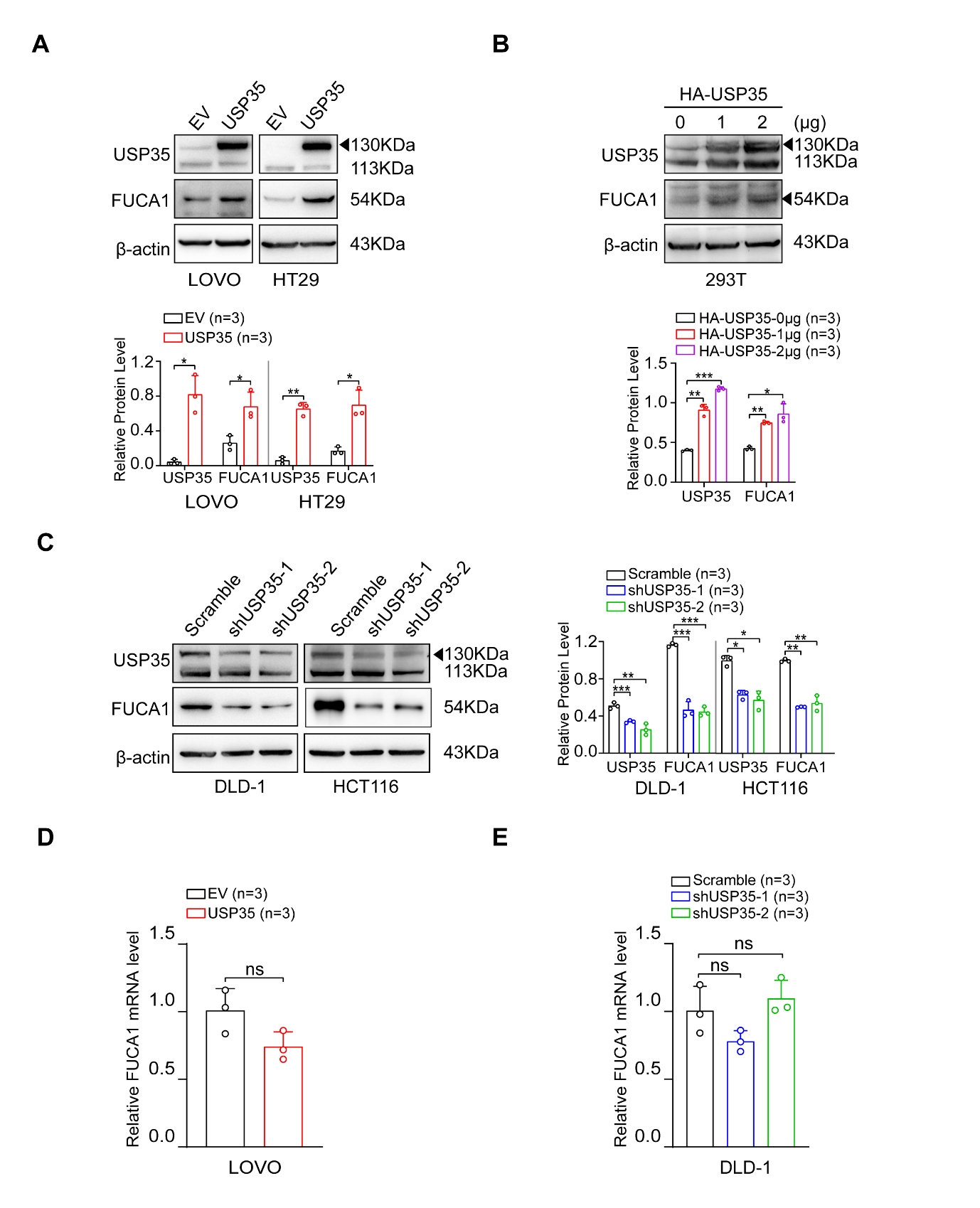
**

**Fig. S6. USP35 stabilizes FUCA1 at protein levels.** (A) Increased expression of FUCA1 at protein levels by USP35 overexpression in LoVo and HT29 cells. Quantitative analysis was shown in the graphs. (B) Dose-dependent increase of FUCA1 at protein levels by different amount of USP35 transfections in HEK293T cells. Quantitative analysis was shown. (C) Reduced expression of FUCA1 at protein levels by USP35 depletion in DLD-1 and HCT116 cells. Quantitative analysis was shown. (D, E) Expression of FUCA1 at mRNA levels remained unchanged by overexpression (D) or knockdown (E) of USP35, as detected by qRT-PCR. Data were presented by mean ± SD of three independent experiments. ns: not significant. *: p<0.05, **: p<0.01, ***: p<0.001 based on the Student’s *t*-test.

**Supplementary Figure S7.**

**
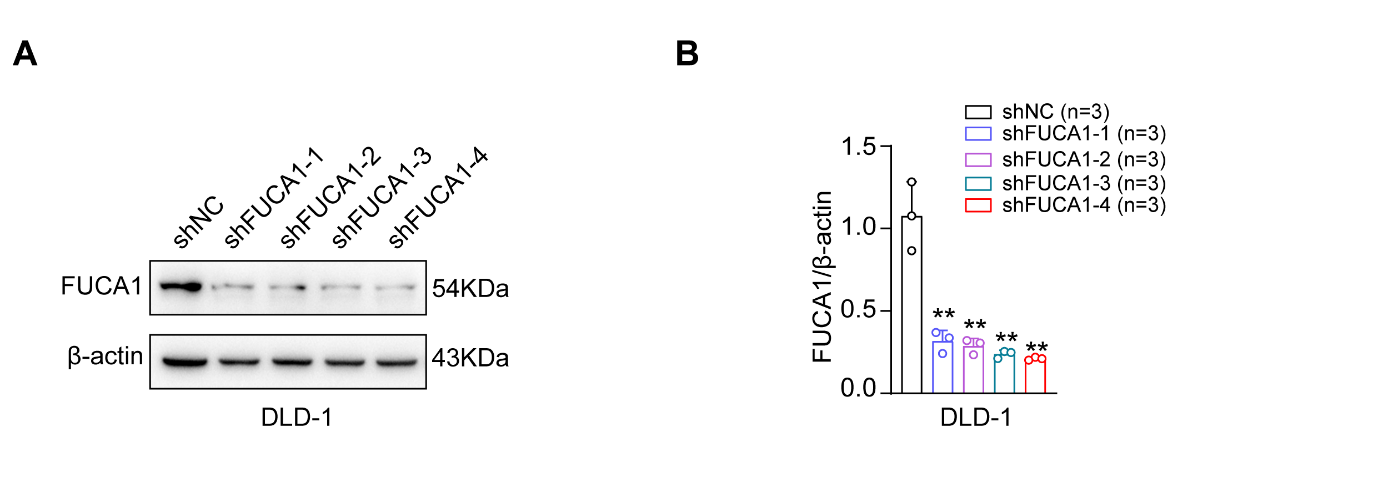
**

**Fig. S7. Knockdown efficiency of different FUCA1-specific shRNAs.** (A) Establishment of FUCA1-depleted DLD-1 cell lines. (B) Quantitative analysis of knockdown rate of FUCA1. Data were presented as mean ± SD of three independent experiments. **: p<0.01 based on the Student’s *t*-test.

**Supplementary Figure S8.**

**
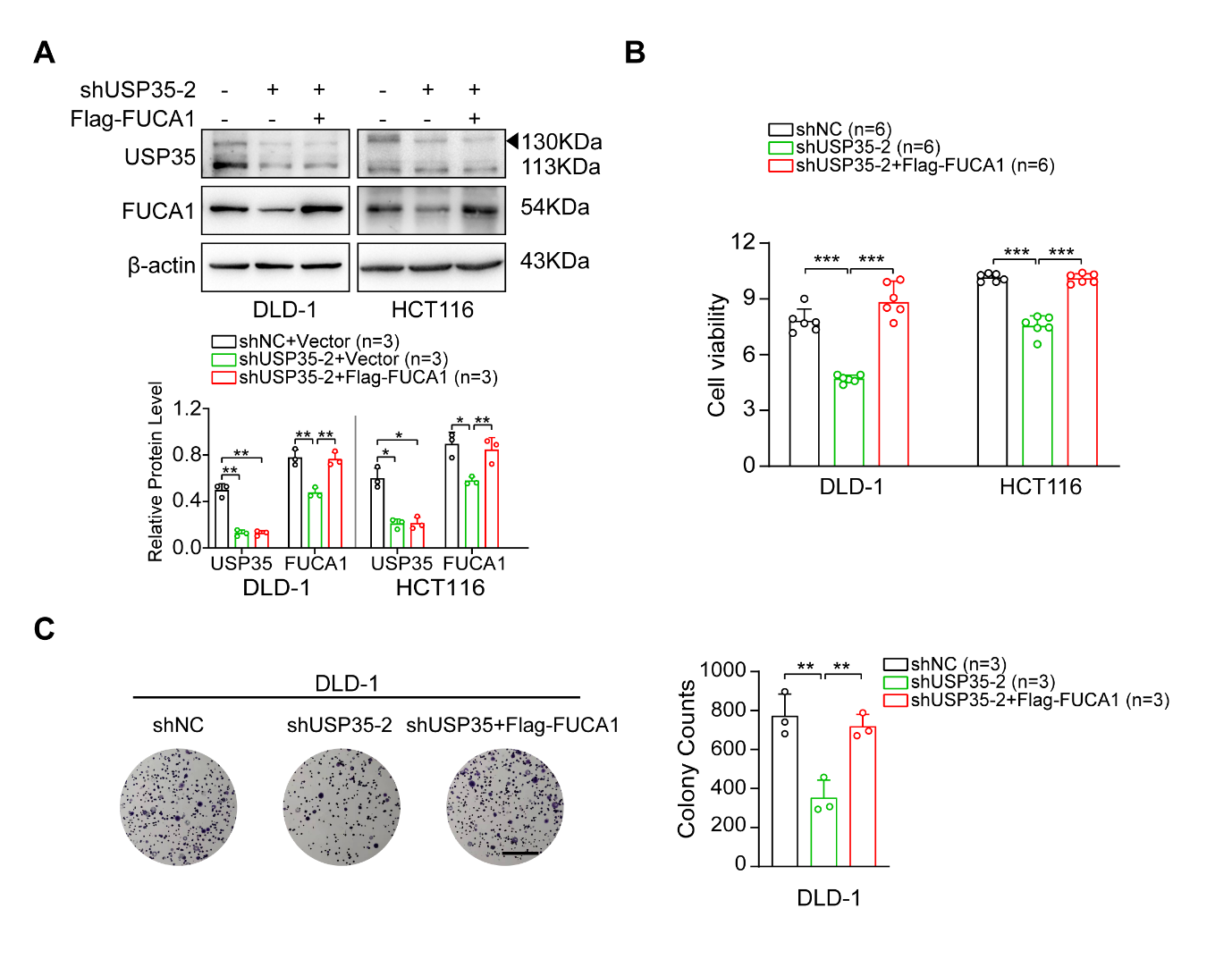
**

**Fig. S8. FUCA1 mediates the effect of USP35 on cell proliferation *in vitro*.** (A) Flag-FUCA1 expression plasmid or control plasmid was introduced into the USP35-silenced (shUSP35-2) DLD-1 and HCT116 cells. The expression of FUCA1 and USP35 was detected by western blotting. Quantitative analysis was shown in the graphs. (B, C) CCK8 assay (B) and clonogenic assay (C) were used to compare the proliferation of FUCA1-overexpressed and USP35-depleted cells with USP35-depleted or control cells. Quantitative analyses were shown in the graphs. The scale bars in Fig. C represented 50 μM. Data were presented by mean ± SD of three independent experiments. *: p<0.05, **: p<0.01, ***: p<0.001 based on the Student’s *t*-test.

**Supplementary Figure S9.**

**
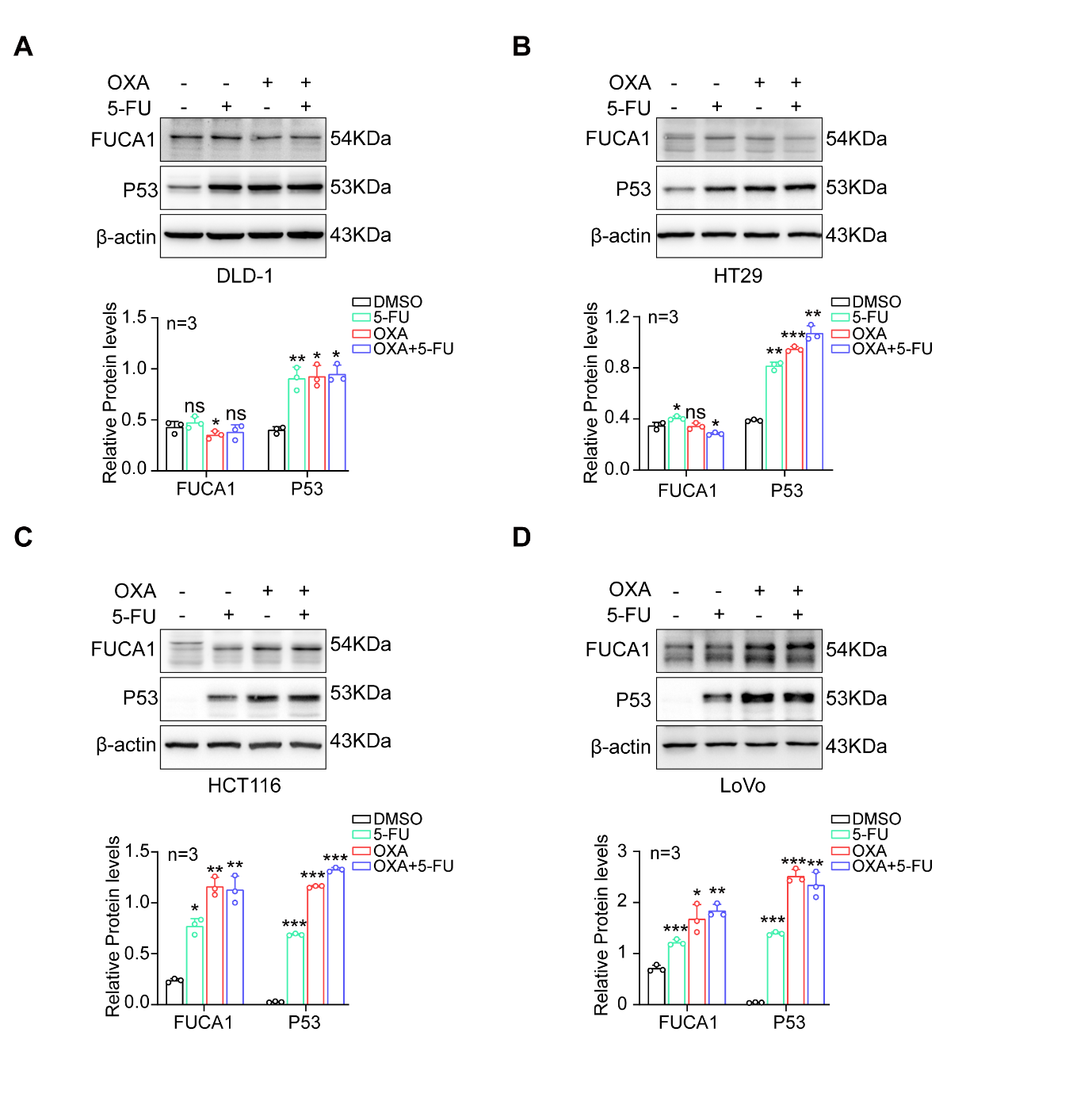
**

**Fig. S9. The effect of the drugs on p53 and FUCA1 expression in CRC cells.** The protein levels of p53 and FUCA1 in DLD-1 (A), HT29 (B), HCT116 (C) and LoVo (D) cell lines treated with or without Oxaliplatin (OXA), 5-FU, or combination of OXA and 5-FU were examined by western blotting assay. Quantitative analyses were shown in the graphs (n=3). All data are presented by mean ± SD. ns: not significant. *: p<0.05, **: p<0.01, ***: p<0.001 based on the Student’s *t*-test.

**Supplementary Figure S10.**

**
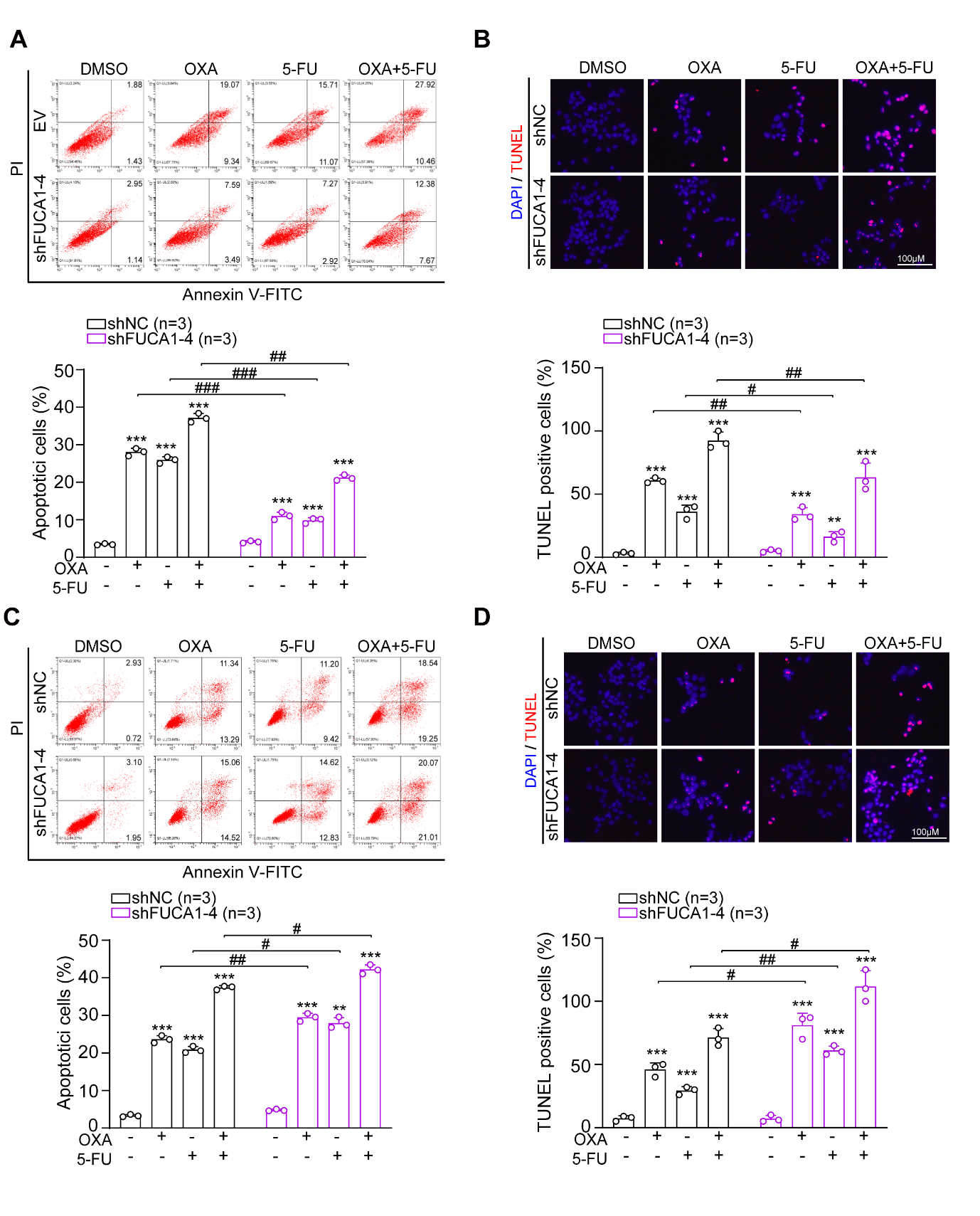
**

**Fig. S10. The effect of FUCA1deficiency on the drug-induced cell apoptosis.** FUCA1-specific shRNA (shFUCA1-4) or control shRNA was introduced into LoVo (A, B) and HT29 (C, D) cells. The cells were treated with DMSO, OXA, 5-FU or combination of OXA and 5-FU for 48 hours (n = 3). Representative images indicated the apoptotic cells detected by flow cytometry analysis (A, C) and TUNEL staining (B, D). The quantitative analyses were shown in the graphs. Data were presented as mean ± SD. **: p < 0.01, ***: p < 0.001; ^#^: p < 0.05, ^##^: p < 0.01, ^###^: p < 0.001 based on the Student’s t-test.

**Supplementary Figure S11.**


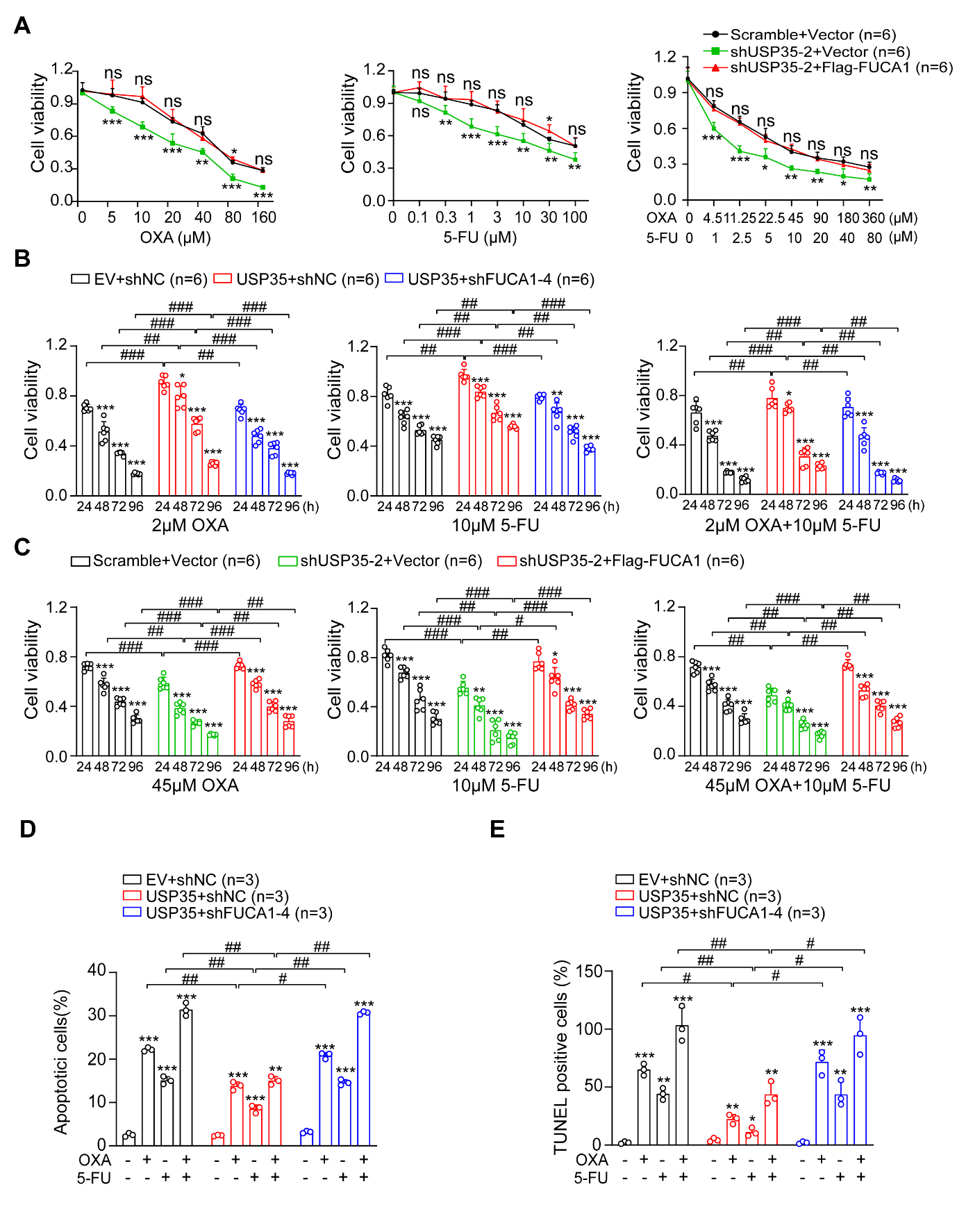


**Fig. S11. FUCA1 mediates the effect of USP35 on chemo-resistance in CRC cell lines.** (A) Flag-FUCA1 expression plasmid or control plasmid was introduced into USP35-depleted (shUSP35-2) DLD-1 cells. The cells were treated with different concentrations of the OXA, 5-FU, or combination of OXA and 5-FU for 48 hours (n = 6). The cell viability was assessed using CCK8 assay. (B) FUCA1 specific shRNA (shFUCA1-4) or control shRNA was introduced into the USP35-overexpressed HT29 cells. The cells were treated with the OXA (10 μM), 5-FU (10 μM) or combination of OXA (10 μM) and 5-FU (10 μM) for 24, 48, 72 and 96 hours (n = 6). The cell viability was assessed using CCK8 assay. (C) Flag-FUCA1 expression plasmid or control plasmid was introduced into USP35-depleted (shUSP35-2) DLD-1 cells. The cells were treated with the OXA (45 μM), 5-FU (10 μM) or combination of OXA (45 μM) and 5-FU (10 μM) for the indicated time points (n = 6). The cell viability was assessed using CCK8 assay. (D, E) Quantitative analyses (n = 3) comparing the apoptotic rate of FUCA1-depleted USP35-overexpressed HT29 cells with USP35-overexpressed cells and the control cells by flow cytometry analysis (D) and TUNEL staining (E) Data were presented as mean ± SD. ns: not significant. *: p<0.05, **: p<0.01, ***: p<0.001, ^#^: p < 0.05, ^##^: p < 0.01, ^###^: p < 0.001 based on the Student’s *t*-test.

**Supplementary Figure S12.**


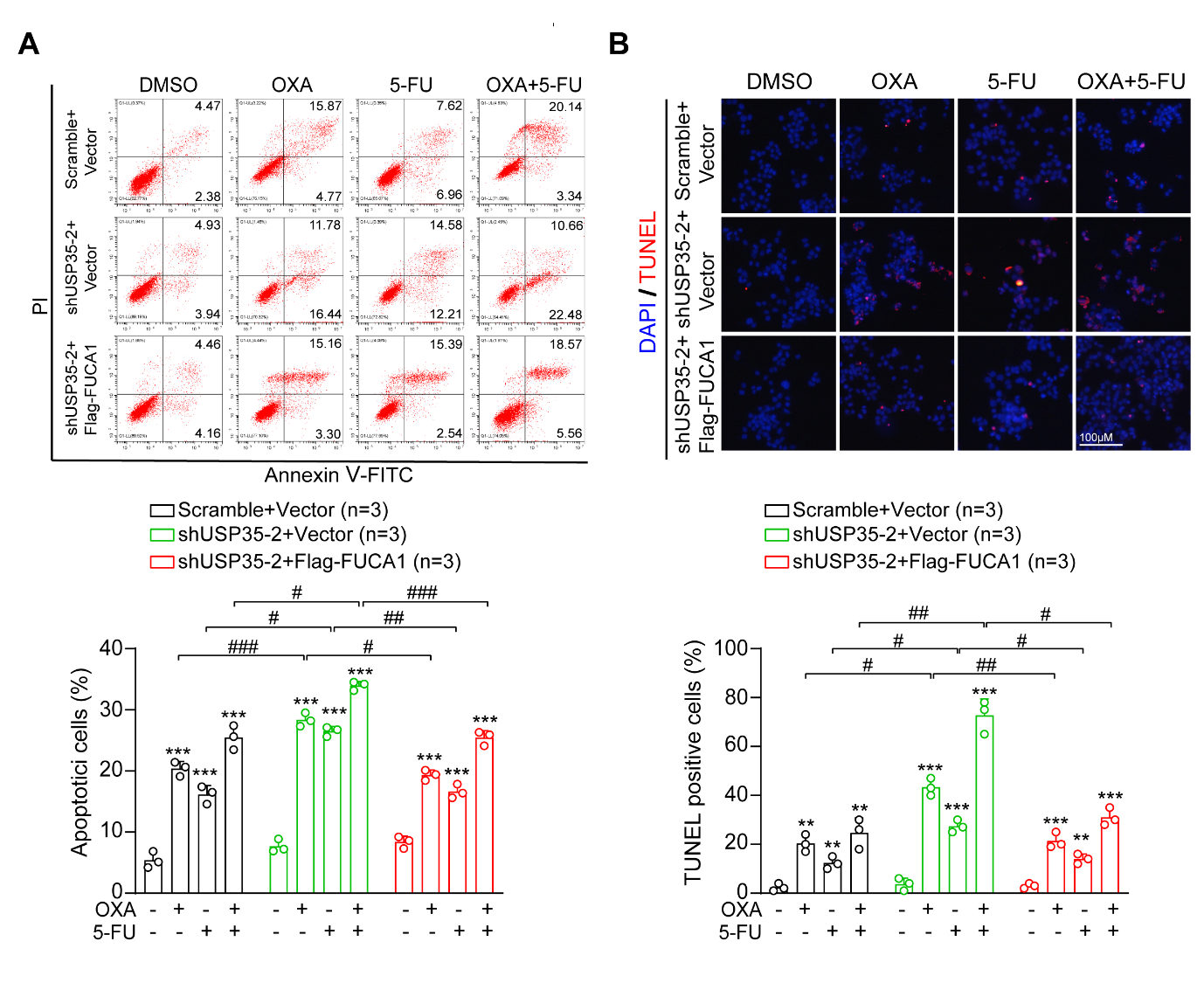


**Fig. S12. FUCA1 mediates the effect of USP35 on chemo-resistance in CRC cell lines.** (A, B) Flag-FUCA1 expression plasmid or control plasmid was introduced into USP35-depleted (shUSP35-2) DLD-1 cells. The cells were treated with DMSO, OXA (45 μM), 5-FU (10 μM) or combination of OXA (45 μM) and 5-FU (10 μM) for 48 hours (n = 3). Representative images showed the apoptotic cells detected by flow cytometry analysis (A) and TUNEL staining (B). Quantitative analyses were shown in the graphs. Data were presented as mean ± SD. **: p<0.01, ***: p<0.001, ^#^: p < 0.05, ^##^: p < 0.01, ^###^: p < 0.001 based on the Student’s *t*-test.

**Supplementary Figure S13.**

**
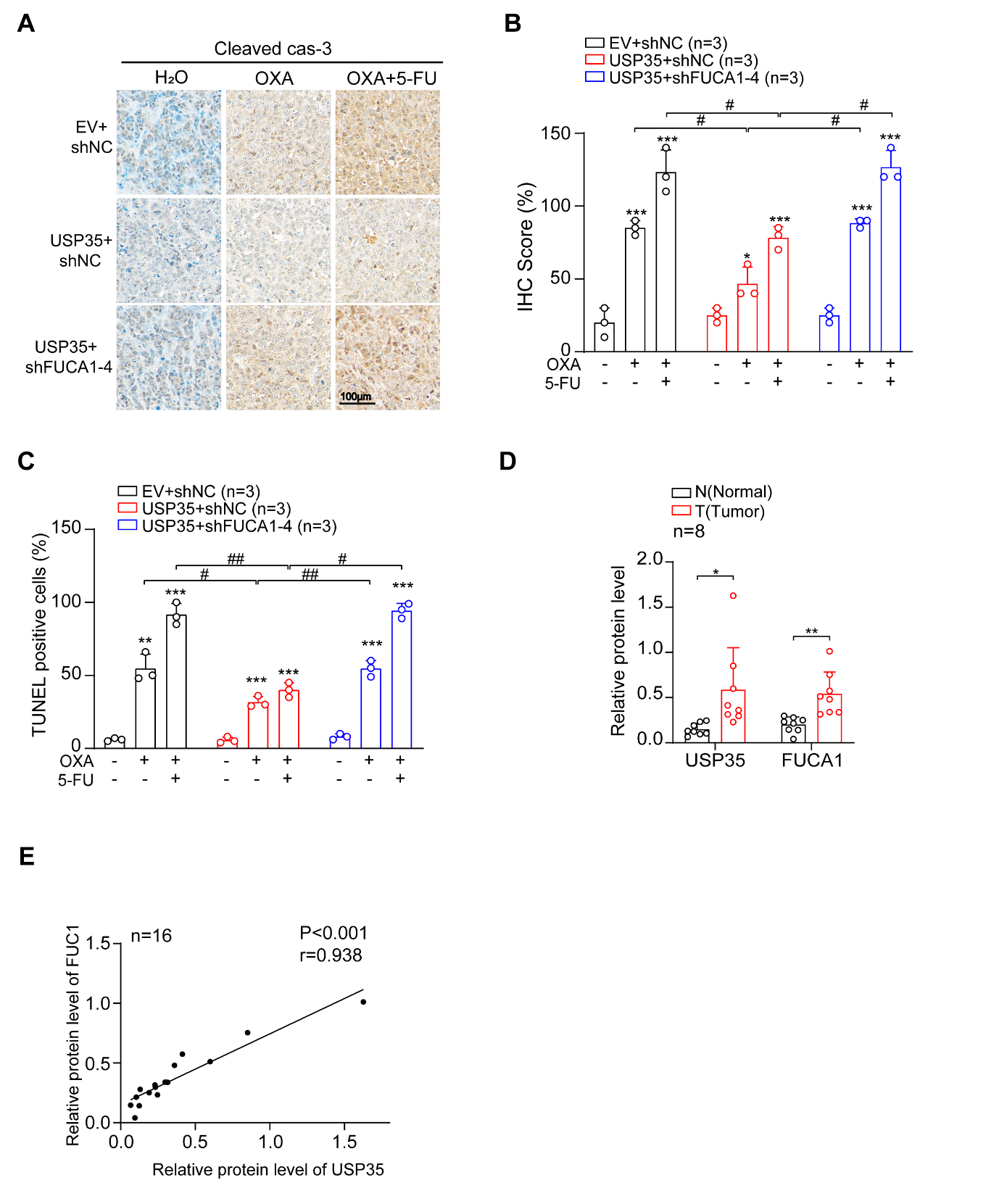
**

**Fig. S13. Correlation of USP35 and FUCA1 expression levels in human CRC tissues.** (A, B) Representative images of Immunohistochemical (IHC) staining (A) and the quantitative analyses (B) of cleaved Caspase-3 in the xenograft samples. (C) Quantitative analyses (n = 3) of TUNEL assay in the xenograft samples. (D, E) Quantitative analysis (D, n = 8) and correlation analysis (E, n = 16) of the expression levels of USP35 and FUCA1 in human CRC tissues and adjacent tissues by western blotting. Data were presented as mean ± SD. *: p < 0.05, **: p < 0.01, ***: p < 0.001, ^#^: p < 0.05, ^##^: p < 0.01 based on the Student’s *t*-test.

**Supplementary Figure S14.**

**
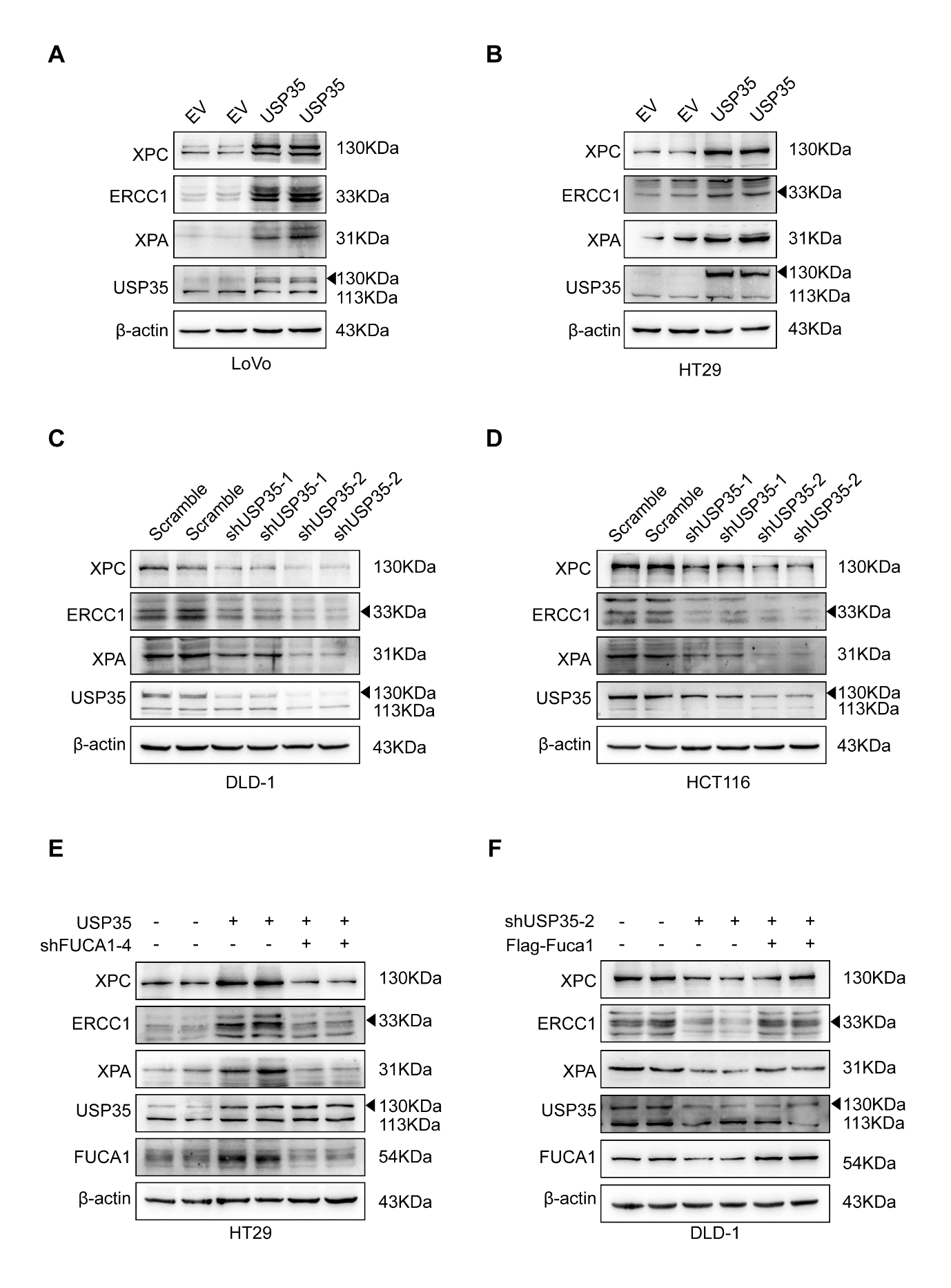
**

**Fig. S14. USP35-FUCA1 axis up-regulates nucleotide excision repair in CRC.** (A, B) Overexpression of USP35 increased the expression levels of XPA, XPC, and ERCC1 in LoVo (A) and HT29 (B) cells. (C, D) USP35 deficiency decreased the expression levels of XPA, XPC, and ERCC1 in DLD-1 (C) and HCT116 (D) cells. (E) FUCA1-specific shRNA (shFUCA1-4) or control shRNA was introduced into the USP35-overexpressed HT29 cells. FUCA1 deficiency reversed the USP35 overexpression-mediated up-regulation of XPA, XPC, and ERCC1. (F) Flag-FUCA1 expression plasmid or control plasmid was introduced into the USP35-depleted (shUSP35-2) DLD-1 cells. FUCA1 augmentation restored the USP35 depletion-mediated down-regulation of the XPA, XPC, and ERCC1. The indicated protein expression was detected by western blotting. All results were the representative of three independent experiments.

**Supplementary Figure S15.**

**
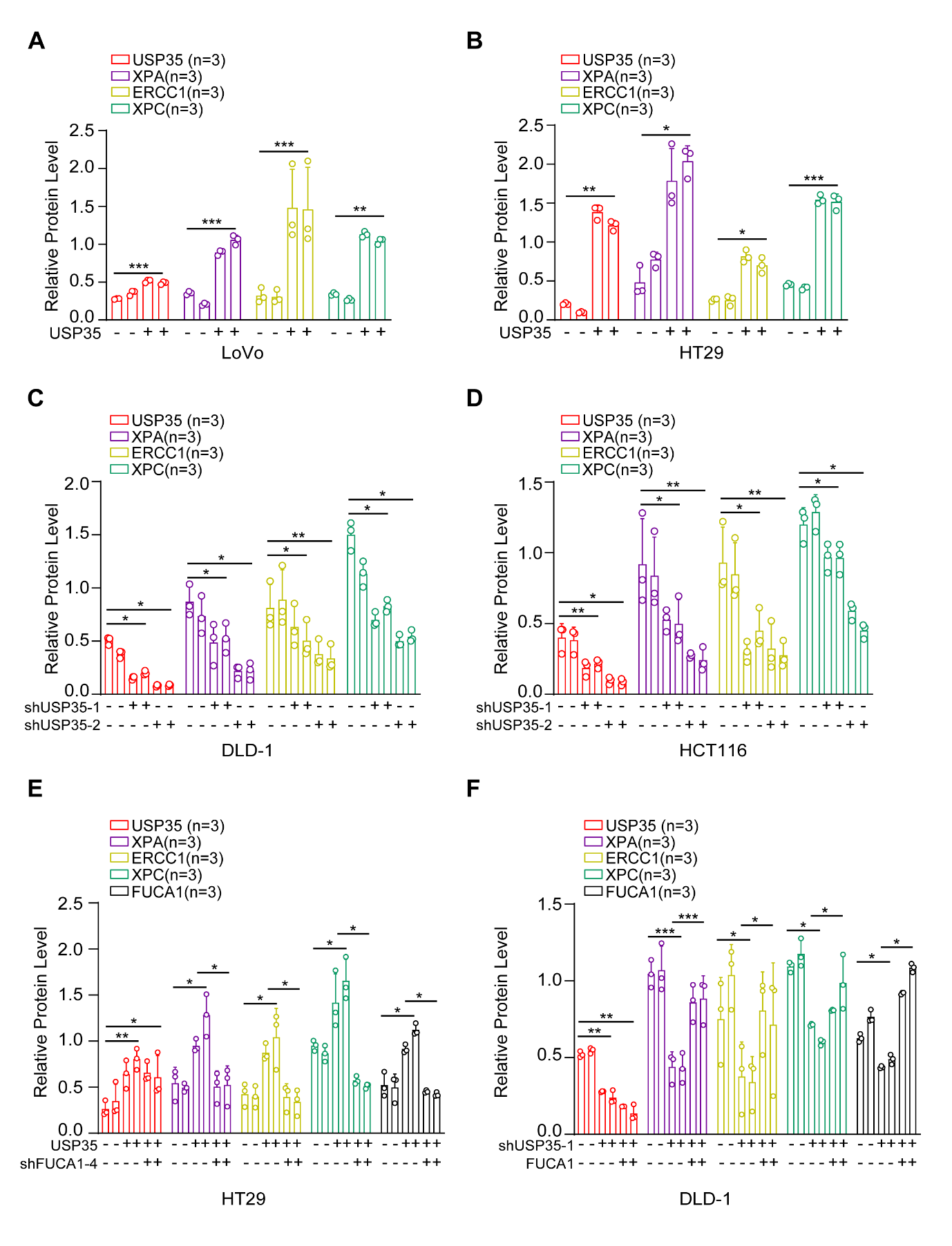
**

**Fig. S15. USP35-FUCA1 axis up-regulates nucleotide excision repair in CRC.** (A, B) Quantitative analyses of the expression levels of XPA, XPC, and ERCC1 by USP35 overexpression in LoVo (A) and HT29 (B) cells. (C, D) Quantitative analyses of the expression levels of XPA, XPC, and ERCC1 by USP35 knockdown in DLD-1 (C) and HCT116 (D) cells. (E) Quantitative analysis showing the results of the indicated protein expression by introduction of FUCA1 shRNA into the USP35-overexpressed HT29 cells. (F) Quantitative analysis showing the results of the indicated protein expressions by overexpression of FUCA1 in USP35-depleted DLD-1 cells. Data were presented by mean ± SD of three independent experiments. *: p<0.05, **: p<0.01, ***: p<0.001 based on the Student’s *t*-test.

**Supplementary Figure S16.**


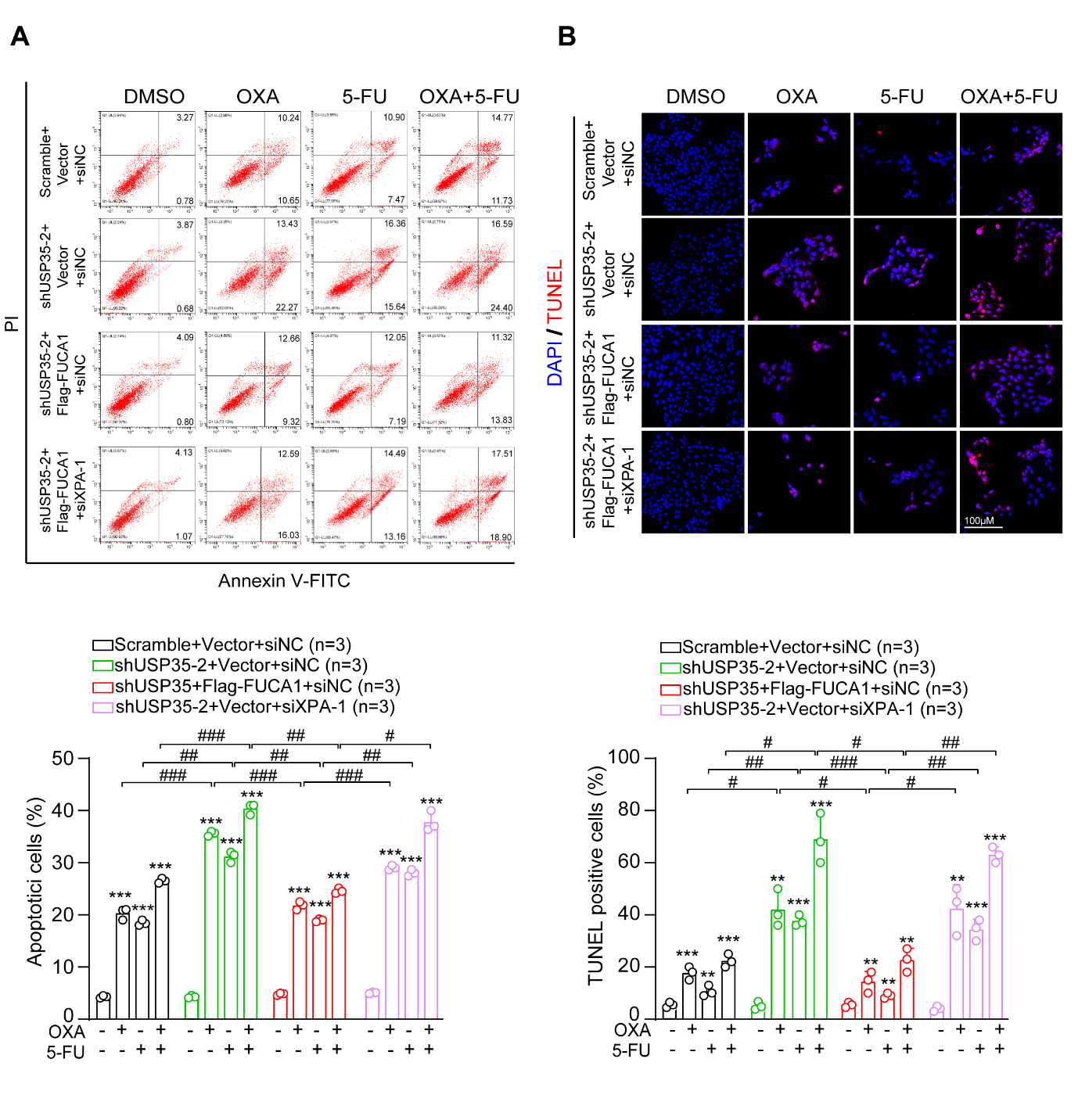


**Fig. S16.XPA is responsible for chemo-resistance regulated by USP35-FUCA1 axis.** (A, B) XPA was silenced in USP35-depleted FUCA1-overexpressed DLD-1 cells by introducing XPA-specific siRNAs and then examined cell apoptosis in response to chemotherapeutics by flow cytometry analysis (A) and TUNEL assay (B). The quantitative analysis was shown in the graph. Data were presented as mean ± SD. **: p < 0.01, ***: p < 0.001; ^#^: p < 0.05, ^##^: p < 0.01, ^###^: p < 0.001 based on the Student’s t-test.

**Supplementary Table S1.**

The stages and mutational status of CRC cell lines.

| Cell lines | APC status | KRAS status | TP53 status | Stages |
| --- | --- | --- | --- | --- |
| HCT116 | Wild type | Muted | Wild type | stage Ⅳ |
| LOVO | Muted | Muted | Wild type | stage Ⅳ |
| DLD-1 | Muted | Muted | S241F | Not available |
| HT29 | Muted | Wild type | R273H | Not available |

**Supplementary Table S2.**

The antibodies used for western blotting and immunohistochemical staining.

| **Antibody** |  | **Working dilution** | **Working dilution** |  | **Species** | | **Source and Cat. Number** |
| --- | --- | --- | --- | --- | --- | --- | --- |
|  |  | **Western blotting** | **IHC** |  |  |  |  |
| USP35 |  | 1:1000 | — |  | | Rabbit polyclonal | Abcam  (Cat. No. ab86791) |
| USP35 |  | — | 1:50 |  | | Rabbit polyclonal | Abcam  (Cat. No. ab128592) |
| USP35 |  | 1:1000 | — |  | | Rabbit polyclonal | Proteintech  (Cat. No. 24559-1-AP) |
| FUCA1 |  | 1:1000 | 1:50 |  | | Rabbit polyclonal | Abcam  (Cat. No. ab230324) |
| XPA |  | 1:1000 | — |  | | Rabbit polyclonal | Sangon Biotech  (Cat. No. D224072-0025) |
| XPC |  | 1:1000 | — |  | | Rabbit polyclonal | ABclonal  (Cat. No. A8354) |
| Cleaved Caspase-3 |  | 1:1000 | — |  | | Rabbit polyclonal | Cell Signaling Technology  (Cat. No. 9664) |
| PARP1 |  | 1:1000 | — |  | | Rabbit polyclonal | Proteintech  (Cat. No. 13371-1-AP) |
| ERCC1 |  | 1:1000 | — |  | | Rabbit polyclonal | Sangon Biotech  (Cat. No. D263621-0025) |
| Myc-tag |  | 1:2000 | — |  | | Mouse monoclonal | Cell Signaling Technology  (Cat. No. 2276) |
| Flag-tag |  | 1:2000 | — |  | | Mouse  monoclonal | Sigma  (Cat. No. F1804) |
| HA-tag |  | 1:2000 | — |  | | Mouse  monoclonal | Sigma  (Cat. No. H3663) |
| Ubiquitin (P4D1) |  | 1:1000 | — |  | | Mouse monoclonal | Cell Signaling Technology  (Cat. No. 3936) |
| β-Actin |  | 1:3000 | — |  | | Mouse polyclonal | Cell Signaling Technology  (Cat. No. ab64659) |
